# Functional profiling of the sequence stockpile: a protein pair-based assessment of *in silico* prediction tools

**DOI:** 10.1093/bioinformatics/btaf035

**Published:** 2025-01-24

**Authors:** R Prabakaran, Yana Bromberg

**Affiliations:** Department of Biology, Emory University, Atlanta, GA 30322, United States; Department of Computer Science, Emory University, Atlanta, GA 30322, United States; Department of Biology, Emory University, Atlanta, GA 30322, United States; Department of Computer Science, Emory University, Atlanta, GA 30322, United States

## Abstract

**Motivation:**

*In silico* functional annotation of proteins is crucial to narrowing the sequencing-accelerated gap in our understanding of protein activities. Numerous function annotation methods exist, and their ranks have been growing, particularly so with the recent deep learning-based developments. However, it is unclear if these tools are truly predictive. As we are not aware of any methods that can identify new terms in functional ontologies, we ask if they can, at least, identify molecular functions of proteins that are non-homologous to or far-removed from known protein families.

**Results:**

Here, we explore the potential and limitations of the existing methods in predicting the molecular functions of thousands of such proteins. Lacking the “ground truth” functional annotations, we transformed the assessment of function prediction into evaluation of functional similarity of protein pairs that likely share function but are unlike any of the currently functionally annotated sequences. Notably, our approach transcends the limitations of functional annotation vocabularies, providing a means to assess different-ontology annotation methods. We find that most existing methods are limited to identifying functional similarity of homologous sequences and fail to predict the function of proteins lacking reference. Curiously, despite their seemingly unlimited by-homology scope, deep learning methods also have trouble capturing the functional signal encoded in protein sequence. We believe that our work will inspire the development of a new generation of methods that push boundaries and promote exploration and discovery in the molecular function domain.

**Availability and implementation:**

The data underlying this article are available at https://doi.org/10.6084/m9.figshare.c.6737127.v3. The code used to compute siblings is available openly at https://bitbucket.org/bromberglab/siblings-detector/.

## 1 Introduction

A typical cell contains about 0.2 g/ml proteins, which translates to up to a billion molecules per cell (Milo 2013, Wiśniewski *et al.* 2014). However, the corresponding number of distinct protein sequences varies from only a few hundred in some bacteria to tens of thousands in many eukaryotes. Characterizing these vital biomolecular nanomachines, i.e. identifying their cellular functions, associated pathways, localization, interaction partners, and catalytic activities, is crucial for understanding their role in cellular biology. Experimental annotation of protein function remains spotty, significantly limited by its cost and speed. For example, among the 94.5 million protein sequences that have been deposited in UniProt in the last three years, only 6974 (<0.01%) were manually curated. Thus, the growing influx of sequencing data has spurred computational annotation of protein function for diverse downstream analyses.

Over the last two decades, the number of bioinformatics tools developed for *in silico* protein annotation has grown and algorithms diversified. Historically, the most common and reliable techniques for annotation relied on the transfer of function by homology, i.e. shared ancestry resulting in sequence similarity. To characterize a given query protein, various alignment and domain profiling tools such as BLAST, PSI-BLAST, and HMMER (Altschul *et al.* 1990, Altschul and Koonin 1998, Potter *et al.* 2018) were used to search annotated protein databases (Mistry *et al.* 2021, Sayers *et al.* 2023, UniProt 2023). Over the years, faster algorithms have been developed to process and annotate consistently growing sequence data, including sequencing reads and genes/proteins extracted from (meta)genome assemblies (Hauser *et al.* 2016, Mahlich *et al.* 2018, Zhu *et al.* 2018, Buchfink *et al.* 2021, Vanni *et al.* 2022). The challenges associated with protein functional annotation are multi-fold and have been discussed at length in earlier studies (Schnoes *et al.* 2013, Mangul *et al.* 2019, Makrodimitris *et al.* 2020, Ramola *et al.* 2022). To summarize the state of the art: aside from exactly defining the word “function” in reference to proteins, there are three bottlenecks in producing accurate annotations—evolutionary caveats that limit function transfer by homology, lack of existing experimental annotations, and limitations of functional ontologies.

The first bottleneck arises as divergent evolutionary processes result in homologous genes of different functions. These could result in false positive functional annotations of sequence- and structurally similar proteins (Todd *et al.* 2002); one such example is the enzymatically inactive duck d crystallin I that shares >90% sequence identity with the active d crystallin II (Sampaleanu *et al.* 2001, Todd *et al.* 2002). At the same time, different genes converging to perform the putatively same function may have minimal homology—a false negative (Todd *et al.* 2001, Gherardini *et al.* 2007); e.g. the human (PDB:1PL8) and *Rhodobacter sphaeroides* (PDB:1K2W) sorbitol dehydrogenases are sequence different. Of course, whether the human sorbitol dehydrogenase is functionally the same as its bacterial version is up for discussion. In general, even those orthologs, i.e. diverged genes found in different species, that do participate in the same molecular mechanisms may not operate at the same rate or efficiency—a functional difference that is often ignored. We argue that context in which the function is carried out should be part of the definition of the function. However, this discussion is beyond the scope of this manuscript.

**Figure 1.**
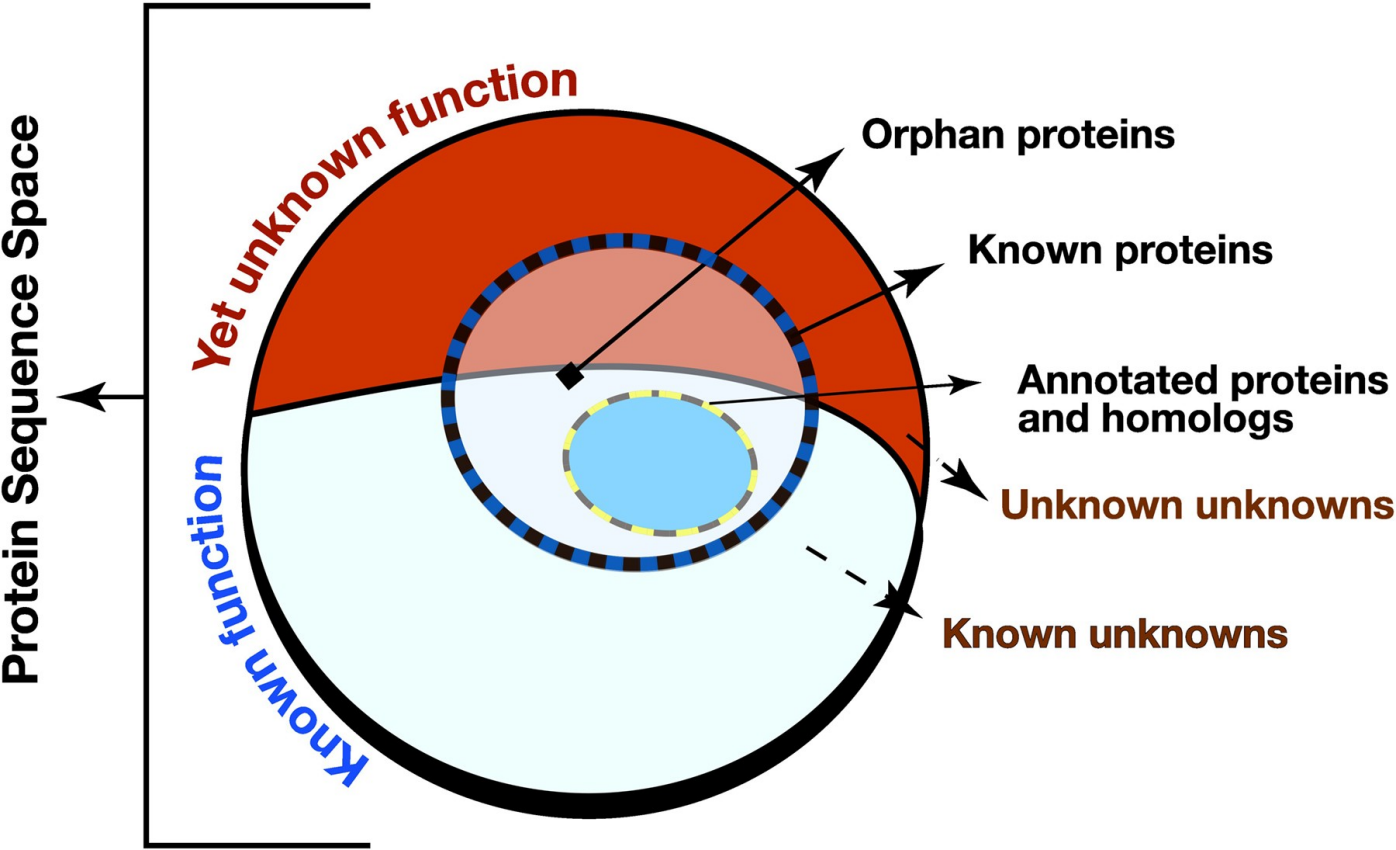
The limits of protein function annotation. Of the complete set of proteins (entire circle), containing known/previously observed proteins (blue/black dashed circle outline) and unknown/not-yet-seen proteins, some fraction carries out unknown functions (fraction of circle in red) rather than known ones (fraction in white). Existing experimental and homology-based protein function annotations (blue oval) cover a small part of the complete protein sequence space. The number of *orphan* proteins, i.e. those lacking annotation and having no known homologs, is growing as we explore our world with better and faster gene/protein capture tools. Note that circle sizes are NOT TO SCALE.

Second, by definition, the general dearth of experimental annotations is limiting for function transfer by homology. Furthermore, existing annotations are biased toward proteins from large families and to species of interest. For example, experimental evidence for GO annotations exists for <15% of proteins in SwissProt (UniProt 2023). The effects of these biases are compounded by computational annotation of newly accumulated genomic data—a process that fosters annotation error propagation. Note that the existing functional annotations only cover the observed part of the protein universe, i.e. annotation of new sequences may be flawed simply due to our limited knowledge of biotic functional capacity (Fig. 1).

The third bottleneck is more technical in nature. The task of representing the ambiguous, environment-dependent, hierarchical role of a given protein with a set of human-understandable ontology terms is exceedingly difficult (Tipton 1994, Ashburner *et al.* 2000, Kanehisa *et al.* 2016a,b, Mistry *et al.* 2021, Saier *et al.* 2021, McDonald and Tipton 2023). Depending on the level of granularity and environmental conditions, a protein’s function could vary widely. For example, all kinases are phosphotransferases that catalyze the transfer of phosphate from ATP to carbohydrates, lipids, or proteins. However, kinases are part of almost every cellular process and many metabolic pathways, i.e. they can be assigned a wide range of biological functions. On the other hand, proteins involved in the same biological pathway have different catalytic (molecular) functions almost by definition; e.g. glycolysis [map00010 (Kanehisa *et al.* 2023)] involves kinases and dehydrogenases. That is, proteins of different molecular functions can contribute to the same biological role, while proteins of the same molecular function may have different biological roles—all across numerous environments and cellular compartments. An ideal protein function ontology should be robust to this variability, but also precise, widely applicable, expandable, and, lately, machine-readable. This ontology does not yet exist.

In short, the classical approach of transferring protein function by homology is complicated by convergent/divergent evolution, lack of experimental annotations, and errors in available computational annotations; it is also limited to existing classes of proteins, reducing chances of discovery of novel functions. A significant amount of research has gone into targeting these challenges to computational function prediction. Novel methods, however, require validation. The Critical Assessment of Functional Annotation algorithms (CAFA) is a community experiment that provides an even ground for their assessment (Radivojac *et al.* 2013, Zhou *et al.* 2019). CAFA uses a time-delayed evaluation where predictions of functions of a large set of yet-to-be-annotated genes/proteins are collected and assessed over a period of time through wet-lab experiments. Research has moved beyond sequence comparison, introducing new computational techniques, and incorporating additional biological data such as the protein-protein interactions, expression, phenotypic changes due to mutation, etc. As such, CAFA results have documented the continuous emergence of new, better-performing prediction methods.

A key recent methodological development was the ability to represent protein sequences as embeddings, i.e. projections of proteins into the latent space. Embeddings are interpretations of deep neural networks, learnt in the process of addressing a predefined objective function (Elnaggar *et al.* 2022, Brandes *et al.* 2022). Protein sequence embeddings have been successful in annotating various protein features, but most obviously protein structure (AlQuraishi 2021, Jumper *et al.* 2021, Lin *et al.* 2023). Function prediction has also been addressed. For example, Littmann *et al.* reported performance on par with CAFA3’s top 10 best performers (Littmann *et al.* 2021, Fenoy *et al.* 2022). Note that besides embeddings capturing protein structural aspects and thus informing function, it remains unclear which aspects of function are reported by these representations.

One important inference from the CAFA experience is the challenge of establishing metrics for the assessment of methods. That is, what is to be considered a correct annotation for a given protein? Given a protein *P*_1_ that carries out functions *f*_1_, *f*_2_, and *f*_3_, if a method *M*_1_ predicts the protein to be of function *f*_1_ only, is this a correct annotation? How does this method perform in comparison to *M*2, which predicts the protein to carry out *f*_3_, *f*_4_, and *f*_5_? While for an individual annotation, say *f*_1_ versus *f*_2_, ontology distance metrics can be established (Wang *et al.* 2007, Clark and Radivojac 2013, Jiang *et al.* 2016, Zhao and Wang 2018), evaluating multiple annotations per protein is harder. Adding to the problem are incomplete annotations, i.e. how would we account for the protein’s unknown functions?

Here, we propose a method and ontology-blind assessment approach for comparison of function annotation tools. We evaluate the predictions of these tools for a set of proteins, sharing little sequence similarity with sequences in available databases. We use structural similarity and a deep learning-based technique to establish whether a protein pair in our set shares functionality, regardless of what specifically they do. We then evaluate other tools’ ability to recall shared functions for these pairs.

## 2 Methods

### 2.1 Extracting the test dataset

From the ESM Metagenomic Atlas (Kulmanov *et al.* 2021), i.e. proteins translated from metagenome records of the MGnify database (Mitchell *et al.* 2020), we collected 53 501 759 protein sequences, translated from metagenome-assembled genes, and having high-confidence predicted 3D structures, i.e. predicted local distance difference test (pLDDT) and predicted TM-score (pTM) >0.9 (Zhang and Skolnick 2004, Mariani *et al.* 2013, Jumper *et al.* 2021). Note that our selected sequences make up less than a tenth of all structures in ESM and represent a significantly smaller fraction still of all metagenome-derived proteins MGnify collected over the years. Thus, the evaluation reported here is limited to a subset of proteins, whose structure is well predicted and, thus, likely biased to reflect that of available, experimentally studied proteins.

These 53.5M sequences were aligned against UniRef100 (Suzek *et al.* 2015) using mmseqs2 (Hauser *et al.* 2016) at default sensitivity = 5.7. Sequences in UniRef are often used as reference for function transfer and as the training set for prediction models (Suzek *et al.* 2015, UniProt 2023). To avoid using model training data in our testing, we focused on 54 359 proteins with <30% sequence identity to UniRef100. For the purposes of this manuscript, we label these proteins *orphans*. Note that this word generally implies a sequence having no detectable homology to known genes/proteins. We suggest that our, somewhat looser definition, provides a simpler identification task for tested methods.

To further simplify the evaluation task, we filtered out proteins over 400 residues-long that are likely to contain multiple domains, as well as proteins whose sequences are truncated in the corresponding MGnify contigs. We further sequence-reduced this set of 11 484 sequences at 90% identity using CD-HIT (Li and Godzik 2006). The final dataset of orphan proteins contained 11 444 sequences with ESM predicted structures and corresponding MGnify cDNA sequences.

### 2.2 SNN + TM: annotating sibling proteins

**Figure 2.**
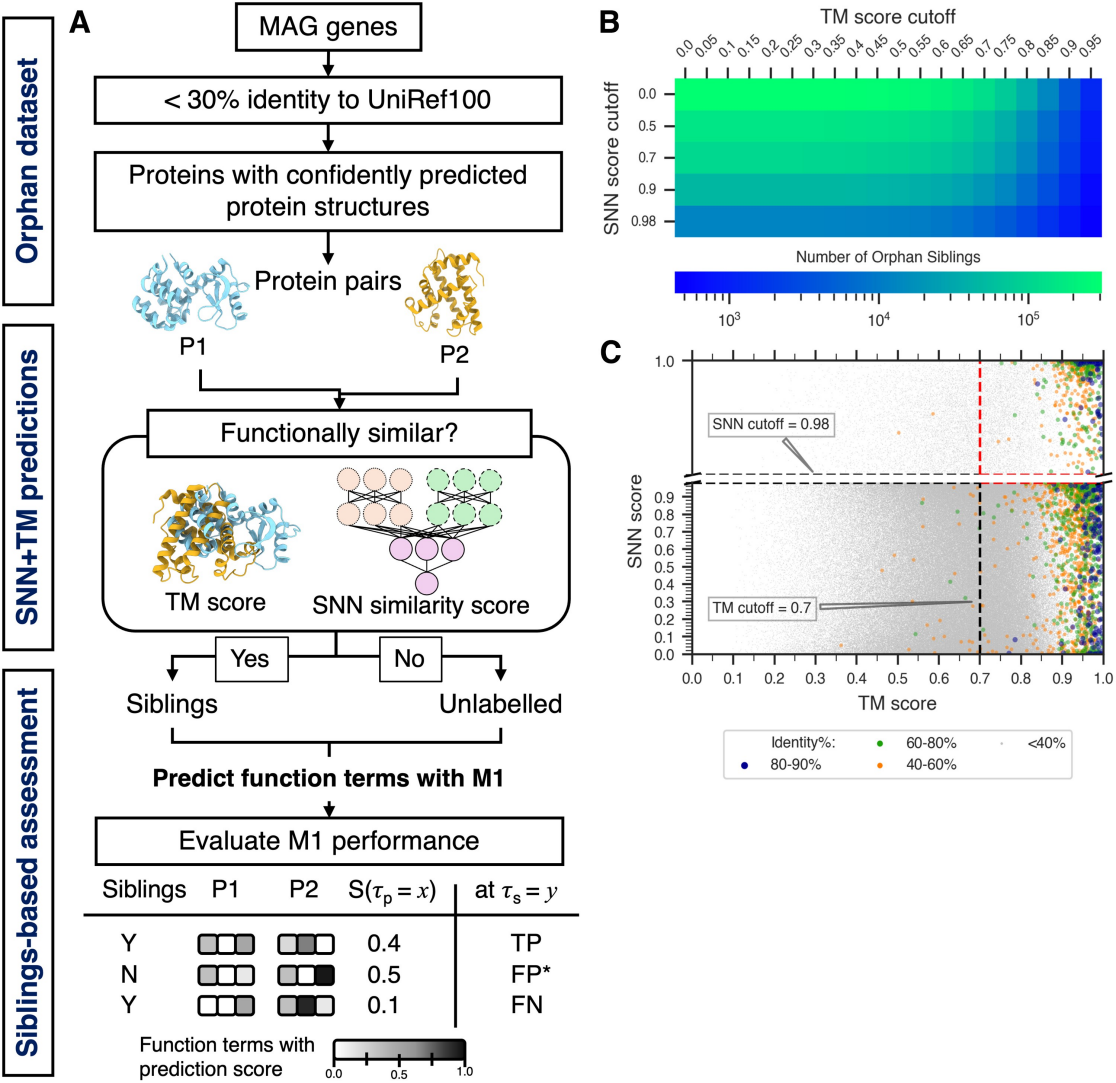
Evaluating function prediction through functional similarity. The performance of function prediction methods is evaluated based on the ability to predict functional similarity between proteins in a pair. (A) Putative functionally similar orphan protein pairs, i.e. a test set of orphan siblings, is built using the SNN+TM method. This SNN+TM test set of orphan siblings consists of protein pairs precisely labeled as siblings [pairs with high structure similarity (TM-score ≥ 0.7) and functional similarity (SNN score ≥ 0.98)] among unlabeled protein pairs. The performance of function prediction methods was evaluated by computing true positives, false negatives, and putative false positives (Section 2). All assessments were conducted by varying thresholds for annotation prediction score (τ_p_) and annotation set similarity (τ_s_). **(B)** The number of orphan pairs considered as siblings at each threshold of TM score (*x*-axis) and SNN score (*y*-axis) in the plot is highlighted according to a log-scale gradient scheme from few proteins (blue) to many proteins (green). In (C) each dot represents a protein-pair and is colored by sequence identity from very low (<40%; gray) to very high (80%–90%; dark blue). Note that no pairs over 90% identity were included in our set. The dashed lines indicate the TM and SNN score cut-offs (0.7 and 0.98) chosen for this work.

In our earlier work (Mahlich *et al.* 2023), we used structural (TM-score ≥0.7) and functional similarity (SNN-score ≥ 0.98), i.e. our SNN+TM approach (Fig. 2A), to identify functionally identical enzyme pairs [same experimentally determined EC number (Tipton 1994)] with 90% precision. SNN is a Siamese Neural Network, trained to identify functionally similar genes (Mahlich *et al.* 2023). The SNN architecture consists of (i) pretrained embedding layer from LookingGlass (Hoarfrost *et al.* 2022), (ii) an LSTM layer, and (iii) the computation of distances between embeddings (Supplementary Fig. S1). TM-scores for protein structure alignments were computed using Foldseek (van Kempen *et al.* 2024).

In our original SNN+TM evaluation, we used available protein PDB structures (Mahlich *et al.* 2023). Here, to find orphan *siblings* (functionally similar proteins) we planned to use the same SNN+TM method, but relying on ESMFold-predicted structures instead (Lin *et al.* 2023). To evaluate the validity of this approach, we extracted from UniProt (2023) 5697 enzymes of length ≤400 residues, annotated with a single, experimentally evidenced EC number. Of these, only 33% (1869) had high confidence ESMFold predicted structures (pLDDT and pTM > 0.9). Trivially, due to the slow growth of experimental data, these enzymes significantly overlapped with those used for the original SNN+TM evaluation. We identified siblings in this set of enzymes and compared sibling annotations to EC pairings. The identified sibling pair was deemed correct if the corresponding EC annotations (at the third level) were the same.

### 2.3 Finalizing the test set pairs

We further extracted functionally similar pairs from our set of 11.4K orphan proteins. First, we compared (Foldseek TM threshold = 0) the predicted structures of all proteins in our set amongst themselves. Only 309 549 of these protein pairs (0.5% of ∼65M possible ones) were structurally similar enough for a complete alignment. We then annotated functional similarity (SNN) scores for these 309K pairs (Fig. 2B). Only 6219 (2% of 309K) pairs attained the pre-set cutoffs (TM score ≥ 0.7, SNN score ≥ 0.98) for shared function and were thus labeled *orphan siblings*. In addition, we explored the performance of the function prediction methods by varying the TM score and SNN score cut-offs (Fig. 4, Supplementary Fig. S5 and Supplementary Table S5), but retained original cutoffs for all future analyses.

### 2.4 Translating predictions into functional similarity

We reformulated the molecular function prediction challenge to overcome the limitations of evaluating and comparing methods that target different functional vocabularies, i.e. tools predicting Gene Ontology (GO) molecular function (MF) terms or Enzyme Commission (EC) numbers. We also included tools that identify protein Pfam domains and assign sequences to KO and COG ortholog groups (Kanehisa *et al.* 2016a,b, Galperin *et al.* 2019, Mistry *et al.* 2021). Note that while GO and EC aim to explicitly describe the function of the protein, Pfam and Ortholog methods capture protein families and evolutionary relationships, which are related to, but not necessary directly reflective of function. If a protein was annotated with multiple Pfam domains, EC numbers, or orthologous groups, we retained all labels. We selected 13 protein annotation tools for our assessment based on the availability of a standalone version or a web server that can process multiple sequences. These 13 methods were grouped based on the type of protein annotation into four categories: GO—DeepFri (Gligorijević *et al.* 2021), DeepGOPlus (Kulmanov and Hoehndorf 2020), GoPredSim (Littmann *et al.* 2021), GOProFormer (Kabir and Shehu 2022), and NetGO (Yao *et al.* 2021), Pfam—HMMER (Eddy 2011), InterProScan (Jones *et al.* 2014), ProtENN & ProtCNN (Bileschi *et al.* 2022) and ProteInfer (Sanderson *et al.* 2023), Orthologs—GhostKOALA (Kanehisa *et al.* 2016a,b), KofamKOALA (Aramaki *et al.* 2020), and E.C.—ECPred (Dalkiran *et al.* 2018) and Mantis (Queirós *et al.* 2021). Note that GOProFormer was developed on yeast proteins (Kabir and Shehu 2022) and using it to predict microbial protein function is beyond its scope of work. Methods such as Mantis (Eddy 2009, Queirós *et al.* 2021) and ProteInfer (Sanderson *et al.* 2023) were included in more than one category since they provide multiple types of protein annotations (Table 1).

**Table 1.** Protein function prediction performance.

Tool	Term	No. of annotated proteins^a^	% of annotatable protein pairs^a^	% of annotatable sibling pair^a^	τ_p_^opt^ at F1_max_^b^	τ_s_^opt^ at F1_max_^b^	ΔS	F1_max_	PR AUC	ROC AUC	Prec	Rec
ECPred	EC1	9402	60.1	73.3	**0.800**	**0.01**	**0.18**	**0.67**	**0.72**	**0.60**	**0.57**	**0.87**
ECPred	EC3	9402	60.1	73.3	**0.890**	**0.01**	**0.25**	**0.68**	**0.70**	**0.67**	**0.65**	**0.75**
Mantis	EC1	1910	8.5	6.3		0.19	0.04	0.64	0.71	0.52	0.51	0.90
Mantis	EC3	1910	8.5	6.3		0.19	0.05	0.61	0.65	0.53	0.52	0.76
DeepFRI	GO	11 328	97.1	98.3	0.214	0.01	0.06	0.47	0.59	0.60	0.62	0.44
DeepGOPlus	GO	11 444	100.0	100.0	0.100	0.02	0.01	0.23	0.40	0.55	0.65	0.15
GOPredSim	GO	11 444	100.0	100.0	**0.384**	**0.05**	**0.14**	**0.63**	**0.66**	**0.61**	**0.57**	**0.72**
Mantis	GO	2895	11.8	12.6		0.38	0.10	0.59	0.60	0.58	0.56	0.64
NetGO	GO	11 444	100.0	100.0	0.087	0.48	0.07	0.53	0.67	0.68	0.62	0.66
ProteInfer	GO	9495	81.3	71.6		0.04	0.02	0.33	0.44	0.56	0.63	0.25
GhostKOALA	KO	3551	7.3	9.7	**0.696**	**0.01**	**0.11**	**0.65**	**0.70**	**0.57**	**0.55**	**0.85**
Mantis	COG	5784	59.9	47.2		0.10	0.04	0.60	0.64	0.53	0.52	0.75
HMMER	Pfam	8661	83.5	76.3	**4.400**	**0.64**	**0.22**	**0.69**	**0.76**	**0.66**	**0.58**	**0.89**
Mantis	Pfam	4874	15.0	26.9		0.37	0.16	0.62	0.65	0.64	0.62	0.65
ProtCNN	Pfam	11 444	100.0	100.0		0.01	0.00	0.13	0.42	0.52	0.77	0.08
ESM	Emb	11 444	100.0	100.0		0.05	0.00	0.19	0.72	0.70	0.25	0.07
LookingGlass	Emb	11 444	100.0	100.0		0.18	0.01	0.35	0.63	0.60	0.62	0.38
ProtT5	Emb	11 444	100.0	100.0		0.08	0.00	0.22	0.72	0.71	0.35	0.12

a Unless explicitly specified, all our performance metrics of each method were computed within the respective subsets of the SNN+TM test set.

b Performance measures at F1 optimal thresholds are computed over 100 iterations of under sampling. Performance of the selected, top-performing methods is identified in bold. Precision and Recall were computed at the F1 optimum prediction score threshold (τ_p_^opt^) and optimum similarity score threshold (τ_s_^opt^). Other performance metrics: ΔS, F1_max,_ PR AUC and ROC AUC are independent of the similarity score threshold (τ_s_) by definition and thus were calculated at the F1 optimum prediction score threshold (τ_p_^opt^).

We also evaluated using pairwise DNA/Protein embedding similarities from unsupervised models such as Bepler (Bepler and Berger 2019), CPCProt (Lu *et al.* 2020), ESM-2 (Lin *et al.* 2023), LookingGlass Encoder (Hoarfrost *et al.* 2022), ProtTrans (Elnaggar *et al.* 2022), SeqVec (Heinzinger *et al.* 2019), and Word2Vec (Dallago *et al.* 2021). In addition, we used SwiftOrtho (Hu and Friedberg 2019), a method that identifies orthologous pairs in a given set of proteins (Chao *et al.* 1992, Dongen 2008), as an upper bound of homology-based evaluation of our test set.

We scored the similarity between predicted annotations of proteins in each pair. Consider a protein pair *P*_1_ and *P*_2_ predicted by method *M*_1_ to carry out sets of functions *Fu*_1_ and *Fu*_2_, respectively. *Fu*_1_ (and *Fu*_2_) consist of several annotation terms from GO, EC, Pfam, or ortholog groups as assigned by *M*_1_; each term is associated with a prediction score (or *E*-value) and is accepted or rejected at a prediction score threshold (*τ*_p_). Increasing *τ*_p_ increases the precision of predicted annotations (*Fu*_1_ and *Fu*_2_) but could also reduce the number of predictions. All performance values reported in this study were computed by varying τ_p_ for methods that provide such prediction scores (Table 1). Note that, at a selected *τ*_p,_ different methods were only able to make predictions for subsets of our SNN+TM test set. Thus, all values reported here, unless explicitly specified, were computed on different sets of protein pairs.

The similarity (*S*) between *Fu*_1_ and *Fu*_2_ is defined by the Jaccard similarity coefficient, i.e. the ratio of the intersection set of terms to the union set [Equation (1)]. In case of GO annotations, we used the information accretion term (Clark and Radivojac 2013, Clark *et al.* 2019) [*I*_a,_  Equations (2) and (3)] to weigh the GO term assignment according to term frequency of appearance among UniProt GO annotations with experimental evidence (Camon *et al.* 2004, Huntley *et al.* 2015); information accretion of a child GO term v, *I*_a_(v), is the information gained by adding v to its parent term(s).

(1) $$S\left(P1\left(\tau_{p}\right),P2\left(\tau_{p}\right)\right)=\frac{\left|Fu1\cap Fu2\right|}{\left|Fu1\cup Fu2\right|}$$

 

(2) $$S_{GO}\left(P1\left(\tau_{p}\right),P2\left(\tau_{p}\right)\right)=\frac{\sum_{v\in Fu1\cap Fu2}I_{a}\left(v\right)}{\sum_{v\in Fu1\cup Fu2}I_{a}\left(v\right)}$$

 

(3) $$I_{a}=-\log\left(\mathrm{Pr}\left(v\right|\mathrm{Parent}\left(v\right)\right)$$

In addition, the similarity score for a protein pair (*P*_1_ and *P*_2_) given their embeddings (*E*_1_ and *E*_2_) of length l, was derived from the Euclidean distance and cosine similarity [Equations (4) and (5)].

(4) $$S_{\mathrm{Euclidean}}\left(P1,\;P2\right)=\frac{0.5}{0.5+\;\sqrt[{2}]{\sum_{i=1}^{l}{\left({E_{1}}^{i}-{E_{2}}^{i}\right)}^{2}}}$$

 

(5) $$S_{\mathrm{Cosine}}\left(P1,\;P2\right)=\frac{E_{1}.E_{2}+1}{2}$$

The performance of a given method in identifying *orphan siblings* was measured first by computing the Area under the Precision–Recall curve (PR AUC), area under the Receiver Operating Characteristic curve (ROC–AUC), and *F*1_max_ by varying the method-specific [Equations (1), (2), (4), and (5)] similarity score threshold ($\tau_{s}$) for calling a protein pair functionally identical. To balance the number of sibling (positives) versus unlabeled (mostly non-sibling) pairs, we under-sampled the latter to match the number of sibling pairs; we repeated the under-sampling 100 times and computed the average and standard deviation of all measures.

To evaluate an “ideal predictor,” i.e. maximum possible performance, we analyzed the annotated enzyme set described above versus the SNN+TM annotations of siblings. Assuming that an “ideal predictor” would produce experimental annotations, 100 iterations of enzyme set balancing resulted in 88% recall and 96% precision (Supplementary Tables S1 and S2). Note that unlike with real function prediction methods that provide prediction confidence scores, here we used binary, i.e. same function versus not, labels inferred from third digit EC number identity between proteins in a pair.

The *F*_1_ score, as the harmonic mean of Recall and Precision, provides an overall prediction performance in identifying siblings [Equation (6)]. We chose the optimum method prediction score threshold ($\tau^{\mathrm{opt}}$_p_) and an optimum similarity score threshold ($\tau^{\mathrm{opt}}$_s_) corresponding to the maximum *F*1 (*F*1_max_); we then reported Precision and Recall at these thresholds [Equations (7) and (8)] for all methods. Note that for methods that predict EC numbers, PFAM domains and ortholog annotations, two proteins were considered to be functionally similar if they shared even one common annotation at a given prediction threshold (τ_p_).

(6) $$F_{1}\;\mathrm{score}=\frac{2\;\left(\mathrm{Precision}\times \mathrm{Recall}\right)}{\mathrm{Precision}+\mathrm{Recall}}$$

 

(7) $$\mathrm{Recall}=\frac{\#\;\mathrm{of}\;\mathrm{correctly}\;\mathrm{identified}\;\mathrm{siblings}}{\#\;\mathrm{of}\;\mathrm{acutal}\;\mathrm{siblings}\;\mathrm{in}\;\mathrm{the}\;\mathrm{dataset}}$$

 

(8) $$\mathrm{Precision}=\frac{\#\;\mathrm{of}\;\mathrm{correctly}\;\mathrm{identified}\;\mathrm{siblings}}{\#\;\mathrm{of}\;\mathrm{predicted}\;\mathrm{siblings}}$$

As described above, our SNN+TM approach has high precision, but a very low recall of functionally similar protein pairs. To compensate for the limitations of thus created test set, we report two additional performance measures: (i) the difference in similarity scores [Δ*S*, Equation (9)] and (ii) the maximum recall while restricting the total predicted positives to fewer than 50% of the data (${\mathrm{Recall}}_{\max}^{\mathrm{PPf}50}$).

(9) $$\Delta S\left(\tau_{p}\right)=\;\mathrm{Recall}*\left(\;\frac{1}{\left|\mathrm{siblings}\right|}\sum_{\left(P_{1},\;P_{2}\right)\;\epsilon \;\mathrm{sibings}}S\left(P_{1},\;P_{2}\right)\;-\;\frac{1}{\left|\mathrm{unlabelled}\right|}\sum_{\left(P_{1},\;P_{2}\right)\;\epsilon \;\mathrm{unlabelled}}S\left(P_{1},\;P_{2}\right)\;\;\right)$$

1. The difference in similarity scores (Δ*S*) between orphan sibling and unlabeled pairs indicates the distance between their score distributions. That is the difference in scores of functionally similar and unlabeled, most frequently not functionally similar, pairs [Eqiation (9)]. We weighted Δ*S* by corresponding methods’ Recall values to penalize methods for failing to identify test set siblings. Δ*S* varies in range [−1,1], where a positive value indicates higher similarity scores for sibling versus non-sibling pairs.
  Note that Δ*S* reflects the distance between siblings and unlabeled pairs in the linear space of similarity scores. As a result, Δ*S* is comparable only among methods with similar similarity-score distributions. Despite its limitations, however, Δ*S* serves as a useful threshold-independent performance measure. Here, we report ${\Delta S}_{\max}$, the highest Δ*S* for each method over the range of prediction score thresholds (τ_p_).
  (10) $${\Delta S}_{\max}=\underset{\tau_{p}}{\max}\Delta S\left(\tau_{p}\right)\;$$
2. We also report the maximum Recall (${\mathrm{Recall}}_{\max}^{\mathrm{PPf}50}$) of each method over the thresholds (*τ*_p_ and *τ*_s_) while restricting the total predicted positives to fewer than 50% of the method-specific dataset. This measure reflects the best possible recall for each method, without encouraging trivial, i.e. “all pairs are siblings” positive overprediction.
  (11) $$\mathrm{Predicted}\;\mathrm{positive}\;\mathrm{fraction},\;\mathrm{PPf}\;\left(\tau_{p},\;\tau_{s}\right)=\frac{\#\;\mathrm{of}\;\mathrm{protein}\;\mathrm{pairs}\;\mathrm{predicted}\;\mathrm{as}\;\mathrm{siblings}}{\mathrm{total}\;\mathrm{number}\;\mathrm{of}\;\mathrm{protein}\;\mathrm{pairs}}$$
   
  (12) RecallmaxPPf50τp, τs=maxτp, τs⁡Recall, if PPf<0.50, other wise 

We compared method performances to two empirical random estimates: a random classifier and a random annotator. A random classifier samples the similarity scores (*S*) of protein pairs from a random uniform distribution. The random annotator is the result of shuffling sibling/unlabeled labels for all protein pairs in the test sets of each method in our assessment. Each simulation was repeated 100 times.

Further, the statistical significance of the performance differences between the tools in terms of Δ*S*, *F*_1max_, PR-AUC and ROC–AUC were assessed through the Wilcoxon rank-sum test and Student's *t*-test along with Benjamini-Hochberg *P*-value correction using SciPy (Virtanen *et al.* 2020).

## 3 Results

### 3.1 Assessing test set and evaluation metrics

We first aimed to evaluate our proposed strategy (Section 2) for assessing protein function annotation methods (Fig. 2). Our protein structure alignment [Foldseek (van Kempen *et al.* 2024)] *plus* shared-function prediction [SNN (Mahlich *et al.* 2023); Supplementary Fig. S1] based approach (SNN+TM; Section 2) captures functional similarity of two proteins, labeling them *siblings*. Structural similarity is often used as a proxy of functional similarity (Kolodny *et al.* 2006, Gherardini and Helmer-Citterich 2008). By filtering SNN predictions to structurally similar proteins, we further assured high precision of our method.

To evaluate our SNN+TM approach (Section 2), we considered 1 745 646 pairs of 1869 enzymes experimentally labeled with an Enzyme Commission (Tipton 1994) (EC) numbers. The TM score component of our method is derived from alignment of the ESMFold (Lin *et al.* 2023) predicted protein structures. The SNN similarity score is predicted by a model that was trained to identify gene pairs encoding proteins from same *fusion* function clusters (Mahlich *et al.* 2023). Note that proteins in different fusion clusters are often sequence similar (homologous), while proteins within the same cluster can be sequence dissimilar. This characteristic of our data results in the observation that our definition of sibling proteins does not specifically reflect protein sequence similarity, i.e. SNN prediction scores for our set of 1.7M enzyme pairs were not correlated (r=−0.036) to sequence identity (Supplementary Fig. S2).

In building our SNN+TM method, we selected the TM and SNN score thresholds (≥0.7 and ≥0.98, respectively) to attain ∼90% precision in capturing functionally similar proteins of our original labeled dataset (Mahlich *et al.* 2023). That is, these cutoffs ensured that most proteins labeled as siblings were correctly labeled, but only a small fraction of all siblings was identified. Thus, for any set of proteins, our approach generated a dataset of positive (sibling) versus unlabeled protein pairs, where the latter could contain siblings but, trivially, significantly fewer of them than non-siblings. Specifically for our set of enzymes, we annotated only 1927 (0.11% of 1.7M) pairs as siblings; of these, 88% (1693) had same EC numbers. On the other hand, of the 1 743 719 unlabeled pairs only 4% (69 042) comprised proteins with same EC number. In other words, our approach attained a recall of 2.4% (1693 of 70 735) and precision of 88% (1693 of 1927).

Going forward in identifying orphan siblings from the unannotated set of proteins we expected the same type of performance. Thus, the test set that we generated for evaluating other methods was of the positive versus unlabeled type. For this type of test sets (Ramola *et al.* 2019, Li *et al.* 2022a,b), the recall of assessed methods, i.e. their ability to identify positives/siblings, is justifiably the primary choice of performance measure. However, to avoid overestimating performance of methods that overpredict positives, we needed to factor in the total number of positives labeled by each method—a measure well captured by precision, i.e. the number of siblings predicted positive versus all positive predictions. We thus decided to use precision here only to illustrate the impact of the total number of positive predictions necessary for each method to recall known siblings.

### 3.2 Orphan siblings as a test dataset

From the MGnify (Mitchell *et al.* 2020) collection of metagenomic data, we extracted a set of 11 444 proteins having <30% sequence identity to any of the UniRef100 sequences (*orphans*) and paired them by expected SNN+TM functional identity (*orphan siblings*, Section 2; Fig. 2). As all existing supervised function prediction methods have been directly or indirectly trained on protein sequences found in UniProt, our approach eliminated any overlap between the training data of the prediction methods and our test set. Thus, our evaluation is an unbiased estimate of functional prediction method performance on any novel proteins.

Of the ∼65M possible protein pairs made from this set, only ∼309K attained a TM-scoreable structural alignment and 6219 pairs (∼2%) were labeled functionally similar orphan siblings by our SNN+TM approach. All other pairs were unlabeled and used in a re-sampling fashion (Section 2) together with the sibling pairs to assess the performance of protein function prediction tools.

Among the 309K protein pairs, 99.3% (307 434) shared <40% sequence identity, while 0.08% (240) were ≥80% identical (Fig. 2C). Of the 6219 orphan siblings, 5576 (89.7%) had <40% identity and 95 (1.53%) were ≥80% identical, i.e. a slight enrichment for sequence similarity among orphan siblings as compared to the complete set of orphans. We note that despite this enrichment, siblings were largely composed of sequence dissimilar protein pairs.

### 3.3 No one method is best for function annotation

**Figure 3.**
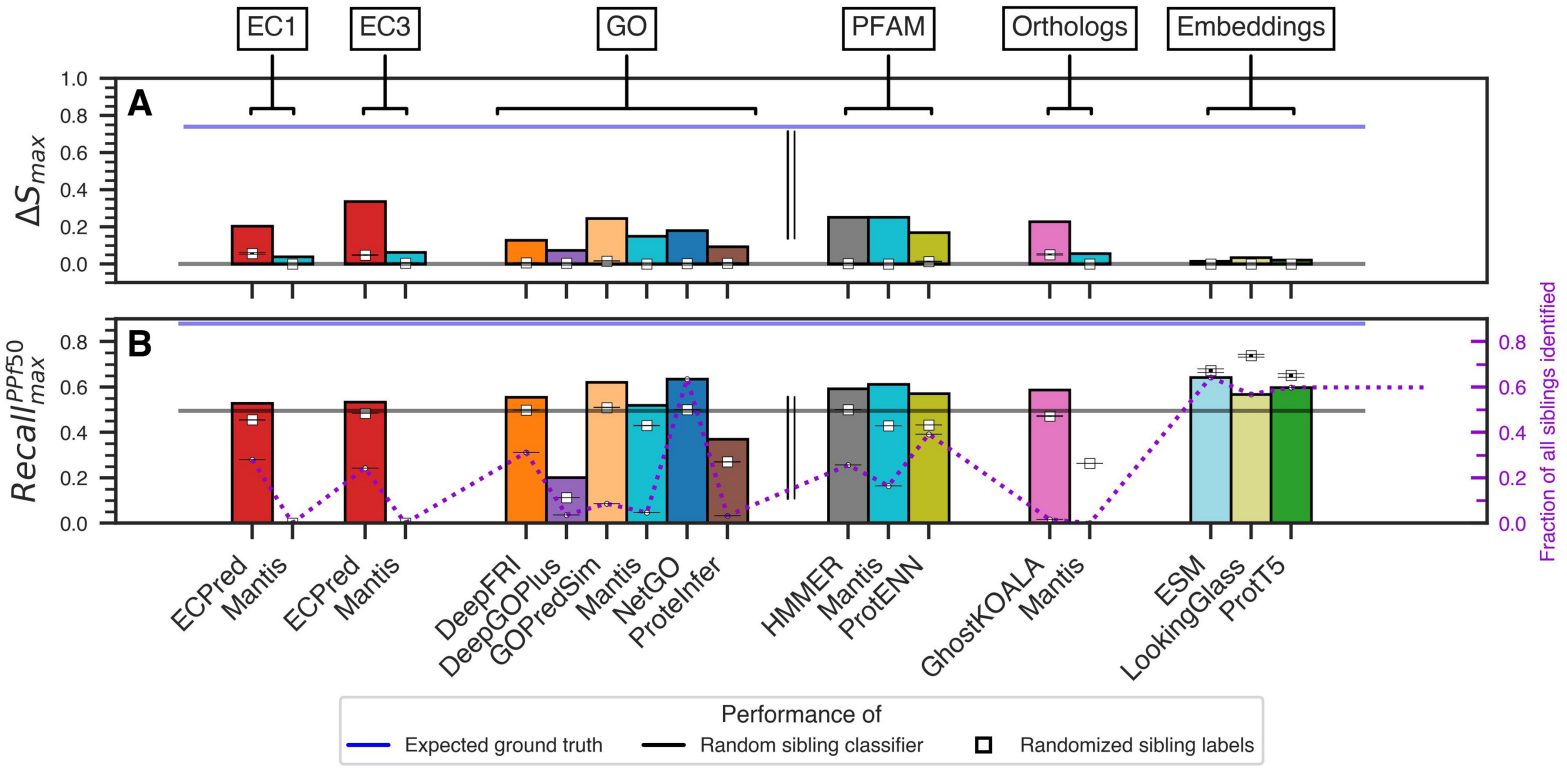
Comparing method performance in each annotation category. The bar plots show the highest (max) per-method (A) ΔS and (B) ${\mathrm{Recall}}^{\mathrm{PPf}50}$ metrics for the SNN+TM test set of protein pairs (see Section 2). The evaluated methods in this study predict: Enzyme Commission numbers (EC1—first digit, EC3—up to third digit), Gene Ontology Molecular Function terms, Pfam domains, and Ortholog groups. Language model embedding distances were also considered. Δ*S*_max_ score was computed for each method by varying the prediction score threshold (*τp*) whereas ${{\mathrm{Recall}}_{\max}}^{\mathrm{PPf}50}$ was computed by varying both the prediction and the similarity score thresholds (*τp* and *τs*). The double line separates explicit functional annotations (EC and GO) from implicit functional annotations via Pfam and Ortholog definitions and embedding similarities. The gray and blue lines indicate the average performance of 100 random baseline classifiers and the expected performance of the “ground-truth” annotation (Supplementary Table S1), respectively. The white squares with standard error bars indicate the average performance of the method-specific random annotators over 100 iterations.

We measured the ability of existing molecular functional annotation methods to assign identical functions to each protein in an orphan sibling pair (Fig. 3, Supplementary Fig. S3 and Table 1; Section 2). Note that methods differed in the number and kinds of proteins they could annotate, resulting in different test sets. Comparing performance of methods across ontologies/prediction vocabularies is inherently a flawed approach due to the differences in (i) completeness of individual functional terminology spaces and (ii) specifics of term distances. However, the top performers in this evaluation were not restricted to any one class of annotation; i.e. ECPred, GOPredSim, Pfam HMMER, and GhostKOALA attained similar performance using the Δ*S*, Δ*S*_max_, *F*1_max_, etc. metrics (Table 1, Fig. 3).

Comparing performance measures derived at fixed thresholds (*τ*_p_^opt^ and *τ*_s_^opt^) may not accurately depict the landscape of method performance. For a more stable measure, we computed the maximum of performance metrics (Supplementary Fig. S3) over a range of both prediction score and similarity score thresholds (*τ*_p_ and *τ*_s_). We also computed *ΔS*_max_, i.e. the linear distance between the distributions of sibling (positives) and non-sibling (negatives) similarity scores—a measure independent of any thresholds (Fig. 3A). Using these metrics, ECPred, GOPredSim, Pfam HMMER, and GhostKOALA retained their position as top performers; in addition, Mantis Pfam predictions attained performance similar to the top scorers. Interestingly, the performance of all methods was somewhat closer to the respective estimates of random than to the expected “ideal” performance (Section 2), highlighting the scope for improvement in function annotation.

We also evaluated the maximum recall (${{\mathrm{Recall}}_{\max}}^{\mathrm{PPf}50}$) of all methods across thresholds (*τ*_p_ and *τ*_s_) while limiting the number of predicted positives to ≤50% of the dataset (Fig. 3B). This constraint restricts the inclusion of low confidence predictions and trivial overprediction of positives. GOPredSim and NetGO outperformed all the other annotation methods in this analysis. However, due to the differences in the number of proteins that could be annotated by each method, NetGO did so for a much larger number of sibling pairs. We thus note that though GOPredSim consistently performed well in all our analyses, the fraction of siblings it identified is significantly lower than other methods (Fig. 3B).

Language models ESM, LookingGlass, and ProtT5, as described below, had similar, ${{\mathrm{Recall}}_{\max}}^{\mathrm{PPf}50}$ as other methods (Fig. 3B and Supplementary Fig. S4). However, the respective performances of their specific random annotators (Fig. 3B) were even higher. Here, we are limited to speculating whether this results from the multi-dimensional and nondiscrete nature of embeddings that encapsulate multiple protein characteristics, including structure similarity, homology, sequence length, etc., instead of function alone.

### 3.4 Protein embedding distance s are not directly informative of functional similarity

We computed similarity between protein pairs based on cosine and Euclidean distances between embedding vectors of all 309K protein pairs [Equations (4) and (5)]. Surprisingly, none of these distances captured the functional similarity between protein pairs well. Using our measures of performance (Δ*S* and ${{\mathrm{Recall}}_{\max}}^{\mathrm{PPf}50}$), none of the language models did better than random. We note that when evaluation uses other metrics, specifically those relying on prediction precision, ESM-2, ProtT5, and LookingGlass embeddings achieve performance similar to some of the better predictors (Supplementary Fig. S4). However, as mentioned earlier, precision is not a reliable metric for our type of positive/unlabeled test data. Further, note that the generic choice of architecture did not drastically differentiate performance–among the top three embeddings, ESM-2 and ProtT5 are transformer-based protein language models (Vaswani *et al.* 2017, Elnaggar *et al.* 2022, Lin *et al.* 2023) whereas LookingGlass is a bi-directional LSTM model trained on short DNA reads (Hoarfrost *et al.* 2022). The other four embeddings were SeqVec (LSTM) and Word2Vec (neural network) inspired by Natural Language Processing (Heinzinger *et al.* 2019, Dallago *et al.* 2021), Bepler is also a bi-directional LSTM trained on amino acid sequences (Bepler and Berger 2019) and CPCProt is a convolution encoder trained through contrastive learning to identify subsequent fragments of protein against random protein fragments (Lu *et al.* 2020).

We also note that given that ESM embeddings could predict protein structures, we expected these to capture functional similarity as defined, in part, by structural alignments. However, interpretation of language models is complicated and the extraction of average representation from multiple layers or a representation of any particular layer is bound to lead to loss of information (Li *et al.* 2022a,b, Hugo *et al.* 2024). Our results thus highlight limitations of cross-domain application of unsupervised deep learning models without extensive analysis and fine-tuning.

### 3.5 Function prediction linked to protein structure

**Figure 4.**
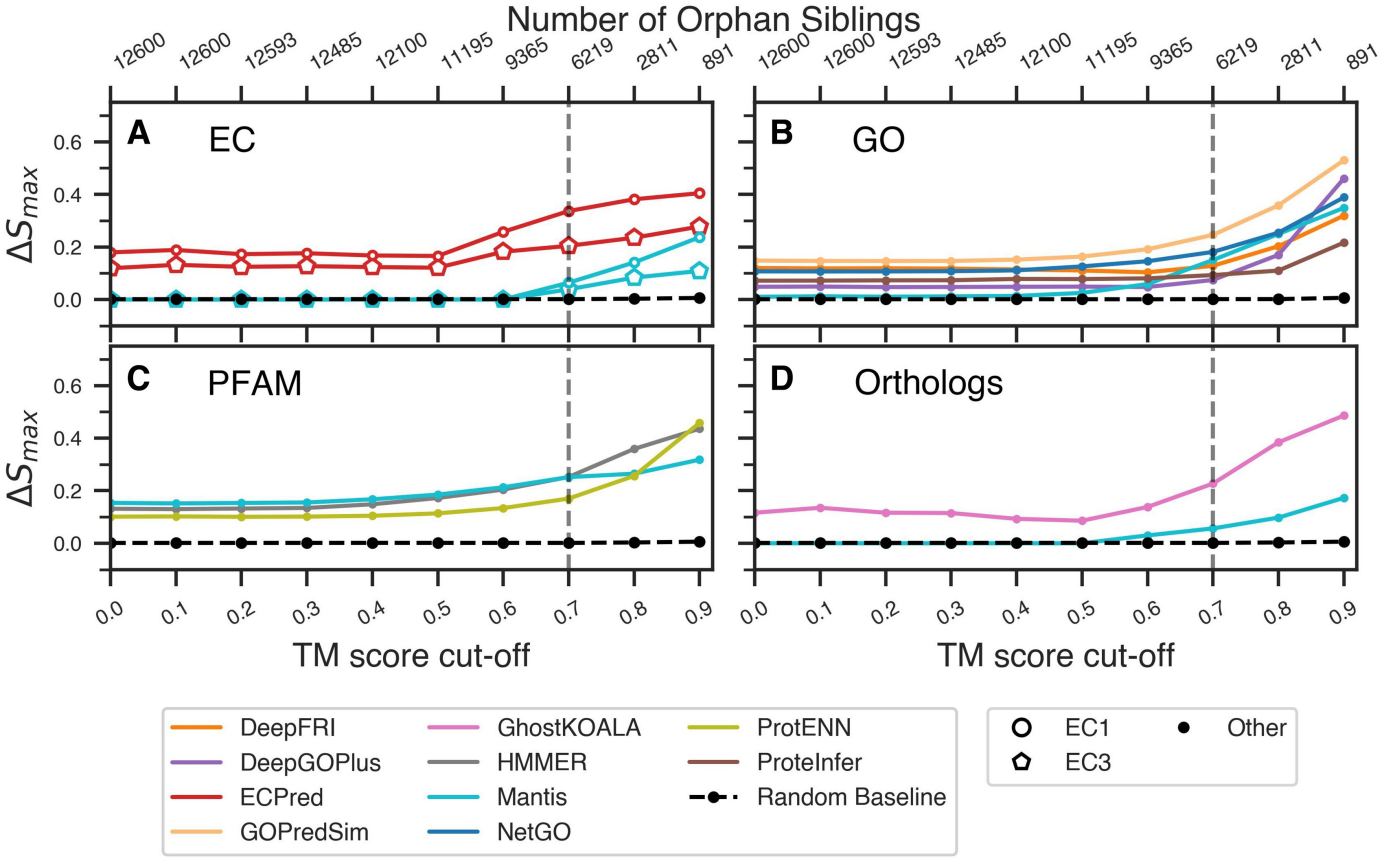
Variations in performance over TM-score cut-offs. The highest (A–D) ΔSimilarity (*y*-axis) of top performing methods vary based on the type of predicted annotations. Note that all scores were computed at SNN cutoff = 0.98 and at different TM-score cutoffs (bottom *x*-axis), resulting in a different number of orphan sibling pairs (top *x*-axis). The average performance of 100 random baseline classifiers is plotted for comparison in each panel (dotted black line). See Supplementary Fig. S3 for trends of *F*1_max_ and AUC *PR*_max_.

Our definition of siblings is dictated by structural similarity (TM alignment) and functional similarity (SNN). By using a high SNN cut-off (0.98), we have negotiated significant reduction in false positives at a loss of true positives. In other words, our approach to identifying functional siblings, while being very accurate (87.8% precision), is known to miss many protein pairs annotated to be of the same function (2.4% recall). While our structural similarity cutoff of TM score ≥ 0.7 is an accepted value (Xu and Zhang 2010) and the SNN cut-off of score ≥ 0.98 was confirmed by our earlier experiments (Mahlich *et al.* 2023), we aimed to explore method behavior across the complete range of protein similarities. We thus computed the prediction performance of methods by varying the TM (Fig. 4) and SNN score (Supplementary Fig. S5) cut-offs and, thus, redefining the protein pairs considered siblings. Note that in evaluating structural similarity we focused on pairs identified by Foldseek as possibly alignable (Section 2), i.e. 309K protein pairs of 65M possibilities, but for these we varied the TM-score in the [0,1] range. The SNN method was trained to recognize pairs as functionally similar above the 0.5 cutoff, so we explored the SNN scores in the [0.5,1] range.

The top-performing methods (ECPred, GOPredSim, HMMER and GhostKOALA) showed consistently better performance than other methods across different TM and SNN score cut-offs. As expected, we observed a steep rise in performance of all methods at TM-score ≥ 0.7 confirming that structural similarity above that threshold plays a significant role in functional similarity. At the same time, restricting the SNN score cutoff to 0.98 increased the performance (AUC under Precision–Recall curve and F1-score) of ECPred, ProteInfer, ProtCNN, and DeepGOPlus by at least 20% (Supplementary Fig. S6) but reduced the performance of GhostKOALA. SNN captures functional similarity independent of either sequence or structural similarity (Mahlich *et al.* 2023). We thus expect that tightening the SNN threshold reduces the performance of methods with strong dependency on sequence similarity, such as GhostKOALA.

Similarly, we repeated our assessment by varying the test dataset. We restricted our analysis to a subset of the test dataset consists of 4376 protein pairs (including 1700 siblings) made up of 3506 proteins with no >150 residues to strictly restrict to single domain proteins (Supplementary Table S4, Supplementary Figs S6 and S7). Overall, we observed a slight in increase in performance of all GO and EC prediction methods except for GOPredSlim. Performance of GOPredSim significantly increased as measured by all the performance metrics, especially Δ*S* which nearly tripled. In contrast, methods predicting PFAM domain and orthologs showed a slight drop in performance. To our surprise, performance of GhostKOALA reduced drastically as observed in the previous observation.

### 3.6 What do the best performing methods capture?

**Figure 5.**
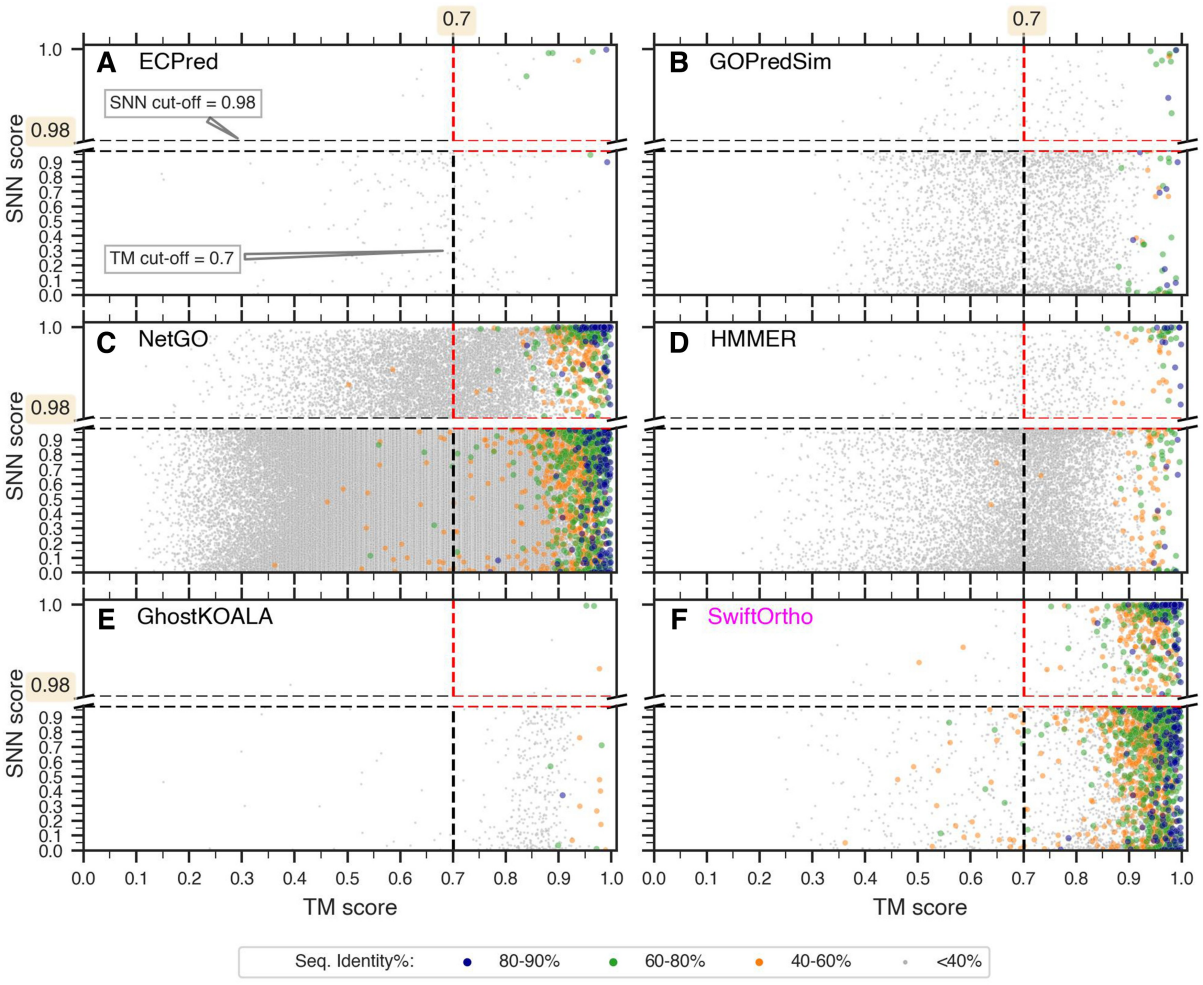
Different annotations capture different functional spaces. Orphan siblings predicted by the top-performing methods (A–E) and SwiftOrtho (F) occupy different spaces in the TM score (*x*-axis) versus SNN score (*y*-axis) space depending on the type of annotation (EC, GO, Pfam and orthologs). Each dot in the plot represents a protein-pair and is colored based on the sequence identity. The dashed lines indicate the TM and SNN score cut-offs (0.7 and 0.98) chosen for the defining the SNN+TM test set of labeled siblings (red dashed lines).

To answer this question, we evaluated contributions of known functionally relevant factors, i.e. sequence and structure similarity, to method functional annotations. We first clustered our orphan proteins, by sequence identity at different cut-offs between 90% and 40% using CD-HIT (Li and Godzik 2006) and explored their shared functionality (Table 2, Supplementary Tables S5 and S6, Fig. 5). We found that less than a tenth of a percent of protein pairs in our orphan dataset set (240 pairs) were highly sequence-similar (80%–90% seq.id), while the vast majority were not; i.e. >99% of protein pairs shared <40% sequence identity. Among all method sibling predictions, we observed significant enrichment of sequence similar protein pairs (≥40% seq. id.) and a depletion of dissimilar pairs. Note that this observation is unrelated to the putative correctness of their functional annotations.

**Table 2.** Characteristics of protein pairs predicted as siblings.^a^^,^^b^

	Range	Total Orphan pairs	Siblings	ECPred	GOPredSim	NetGO	HMMER	GhostKOALA	SwiftOrtho
Sequence Identity	<40%	307 434 (99.3%)	5576^-^ (89.7%)	219^--^	3724^--^	200 223^--^	10 696^--^	456^--^	2150^--^
	40%–60%	1155 (0.4%)	314^++^ (5.0%)	1	11	1131^++^	88^++^	9^++^	1019^++^
	60%–80%	720 (0.2%)	234^++^ (3.7%)	5^++^	32^++^	714^++^	58^++^	6^++^	717^++^
	80%–90%	240 (0.1%)	95^++^ (1.5%)	2^+^	10^+^	237^++^	19^+^	1	239^+^
TM-score	0–0.5	39 675 (14.5%)	0^--^	22^-^	301^--^	25 666^--^	891^--^	10^--^	118^+^
	0.5–0.7	133 206 (45.0%)	0^--^	111	1547^--^	88 625^--^	4039^--^	15^--^	249^--^
	0.7–1.0	136 668 (40.4%)	6219^++^	94	1929^++^	88 014^++^	5931^++^	447^++^	3758^++^
SNN score	0–0.5	102 620 (50.1%)	0^--^	88^--^	1970^+^	98 533^--^	5726^++^	298^++^	1436^+^
	0.5–0.98	181 495 (45.8%)	0^--^	120^+^	1644^-^	93 997^++^	4584^--^	167^--^	1841
	0.98–1.0	25 434 (4.1%)	6219^++^	19^+^	163	9775^++^	551^++^	7^-^	848^++^

a Values indicate the number of siblings predicted by each method in the given range of sequence identity, TM-score, or SNN score. The values were compared against the corresponding counts in the entire dataset of orphan pairs via the two-sided Fischer’s exact test.

b +/- denotes a significant increase/decrease with *P*-value in [0.001, 0.05] range, while ++/-- denotes *P*-value of <0.001.

We further observed that the enrichment of GOPredSim and ECPred sibling predictions was limited only to pairs of higher similarity (≥60%). GhostKOALA’s predictions, on the other hand, were not significantly enriched in highly sequence similar pairs (80%–90%). Note that the small number of these highly sequence similar pairs complicates inference. That is, of the 240 such pairs, GOPredSim and ECPred identified ten and two, respectively—a small, but significant number—while GhostKOALA, which is built to annotate proteins using ortholog information, identified only one. We expect that the latter result is due to our test set being made up of orphan proteins, i.e. those without homologs in the predictor’s reference. These observations suggest that, as expected, sequence information is important in driving function annotation by all methods, but GOPredSim and ECPred are more reliant than others on high sequence similarity.

The enrichment in the number of siblings predicted by each method within sequence-similar bins did not correlate with higher function prediction accuracy across these bins. For example, ECPred precision was worse for high similarity pairs than for lower ones, i.e. the opposite of the enrichment trend (Table 2 and Supplementary Table S6). On the other hand, GOPredSim precision was similar for all sequence identity bins. HMMER which showed significant enrichment in the 40%–80% sequence identity bin, had the highest precision of 63% in the 80%–90% identity space. To summarize, while the predictions of the methods in this study are biased toward identifying sequence similar proteins as siblings, the accuracy of such predictions in terms of their functional similarity does not agree with this assessment.

We also explored this homology-based functional annotations using SwiftOrtho (Hu and Friedberg 2019)—a method that identifies orthologous pairs in a given set of proteins based on sequence similarity(Chao *et al.* 1992, Dongen 2008). As expected, SwiftOrtho correctly identified 239 of 240 of the highly sequence similar (80%–90% sequence identity) pairs; its predictions were also enriched in pairs of sequence similar proteins at all levels of similarity ≥40% (Table 2 and Supplementary Table S5). These results highlight the success achievable by homology-based methods in the presence of the relevant reference sets and further emphasize their deficiency in the absence of such reference. We note, however, that distinguishing orthologs from paralogs is hard (Nehrt *et al.* 2011, Gabaldon and Koonin 2013) and even harder without the taxonomic and/or genomic context. In fact, SwiftOrtho also (putatively) incorrectly labeled 144 protein pairs as siblings.

We further analyzed our data by binning predicted siblings based on structural (TM-score) and putatively functional similarities (SNN score). Predicted siblings from all methods, except ECPred were enriched in high structurally similar pairs (TM-score=[0.7,1.0]) versus the low similarity range (TM-score=[0,0.5], Table 2). In other words, while most methods capture functional similarity driven by structural similarity, ECpred identified similar enzymatic activity in remotely structurally similar protein pairs as well. Note that ECPred predictions were significantly enriched in the high SNN score space ([0.98,1]). This is not unexpected given that functional convergence is more probable than sequence or even structural convergence (Doolittle 1994, Almonacid *et al.* 2010) and ECPred relies on (sub)sequence and physiochemical feature similarity to predict EC numbers (Dalkiran *et al.* 2018).

Different patterns yet were observed in bins reflecting moderate levels of structural similarity (0.5–0.7) and sequence identity (40%–60%). GhostKOALA showed a 5-fold enrichment in protein pairs with moderate levels of sequence identity and a 14-fold depletion in protein pairs with moderate levels of structural similarity; a similar trend was observed for HMMER. Note that protein pairs with a TM-score over 0.5 are highly likely to share fold-level similarity (Xu and Zhang 2010)—a feature that can be expected to reflect function, but does not appear to be useful to the methods reported here.

To summarize, sequence similarity is widely recognized as a key determinant in assessing functional similarity, particularly due to many functional evolution events resulting from gene duplication (Taylor and Raes 2004). However, we hypothesize that existing protein sequence and domain recognition-based methods are biased toward capturing sequence similarity over functional signatures, thus failing to capture analogous evolution, reflect on function diverged between sequence-similar homologs, and identify conserved function among highly diverged siblings (Galperin *et al.* 1998, Todd *et al.* 2001, Omelchenko *et al.* 2010).

Even considering the incomplete and erroneous functional annotations, everything we currently know about specific proteins and their functions is only a minor fraction of the entire protein universe (Ghatak *et al.* 2019). However, annotating new proteins based on available data seems to be an inherently flawed proposition. Of the 53 million high-quality predicted ESM structures of microbial proteins extracted from MGnify only 54K (0.1%) had <30% sequence identity with UniRef. In turn, the overlap between all of UniProtKB and MGnify is estimated to be <1% (Mitchell *et al.* 2020). That is, quality structure predictions, even with the aid of protein Language Models (pLMs), are limited to known protein families restrained by homology. For orphan proteins, this could explain the lacking performance when using embeddings (Fig. 3, Supplementary Figs S3 and S4). Nevertheless, GoPredSim which leverages function-transfer based on embedding similarity through k-nearest neighbors, is one of our four top performers, underscoring the potential of adapting current deep learning techniques to identify functional similarity among proteins.

## 4 Summarizing the findings

With the growing stockpile of sequences, development of accurate functional annotation tools is more essential than ever. A major limitation to robust assessment, and development, of protein annotation tools is the lack of large and diverse “ground truth” annotations. In this work, we translate the challenge of function prediction into a task of identifying functionally similar protein pairs. We use this alternate approach to assess performance of protein annotation tools on a set of “orphan” proteins, i.e. those that have no known close homologs. Careful evaluation across a range of metrics reveals that even the performance of the top methods (ECPred, GOPredSim, HMMER, and GhostKOALA) on these orphan proteins is somewhat lacking.

We note that even though the methods considered here use different annotation vocabularies, as long as their annotations reflect molecular function, our approach of deriving protein similarity scores still allows for assessment of their performance. However, performance comparison across different ontologies is limited by the mutual coverage of functional space. For example, the entire collection of EC numbers is covered by only ∼40% of GO terms (4908 terms). Furthermore, while we use distance metrics to evaluate prediction similarity, the inherent ontology structures likely also have an impact on performance measures. As such, we do not suggest comparing method performance across the ontology spaces.

In this review, we also explored the definition of protein functional similarity in terms of sequence and structure. Machine learning-based models such as ECPred, NetGO, and GOPredSim capture more than sequence similarity from input sequences, unlike the more sequence homology-based algorithms. However, functional similarity is not only a function of sequence or structural similarity. The robustness of protein conformations paves way for diverse or similar sequences fold into diverse or similar structures to carry out the same or different functions as the environment dictates.

Another key observation from our work is that there is a lot of room for improvement in training deep learning models for protein functional annotation. Neither specifically trained methods, nor the direct application of protein embeddings to identify functionally similar pairs showed promising results, highlighting the need for fine-tuning and analysis. While a huge advance has been made in protein structure determination in recent years, similar improvement in function annotation is limited by the lack of ground-truth annotation. However, alternate approaches to evaluation, such as the one we put forward, could pave the way for better models.

We note that despite the advantages and broad application scope of the proposed approach, our ability to label functionally similar protein “sibling” pairs is limited to computation assessment. We used the structural similarity (TM score), combined with deep learning-based functional similarity predictions (SNN), to define these siblings with high confidence but at the cost of low recall. We thoroughly explored the implications of the specifics of our approach by varying the TM score and SNN score thresholds. Notably, the key findings reported in the manuscript remained consistent across these assessments and performance evaluations did not obviously favor any subset of tools under consideration. We believe that our future work on identifying protein sibling annotation will resolve the recall limitation. In particular, applying our annotation assessments may lead to synergistic development of prediction methods and evaluation techniques.

While our test set can help in evaluating functional annotation tools, it can not represent the “ground truth” for the function prediction problem. Here, the only other possible source of data for evaluation is novel experimental annotations of proteins. Nevertheless, we believe that using these data, as in the CAFA initiative, would still benefit from the “sibling”-based perspective to performance evaluation.

## Supplementary Material

btaf035_Supplementary_Data

## Data Availability

All data used in this study are listed in the main text or deposited in a permanent online data repository. The dataset of orphan proteins and the function similarity scores are available at 10.6084/m9.figshare.c.6737127. The code used to compute siblings is available openly at https://bitbucket.org/bromberglab/siblings-detector/.
