# Supplementary material for: Functional profiling of the sequence stockpile: a protein pair-based assessment of in silico prediction tools
Source: Bioinformatics. 2025 Jan 24;41(2):btaf035. doi: 10.1093/bioinformatics/btaf035 (PMC11821270; doi:10.1093/bioinformatics/btaf035)
Supplement: btaf035_Supplementary_Data [file btaf035_supplementary_data.zip › 4c088_FuncPred22Rev-SOM_tables_figures_R1.pdf]

Functional profiling of the sequence stockpile: a protein pair-based assessment of *in silico* prediction tools

Prabakaran R[1,2*], Bromberg Y[1,2*]

1 Department of Biology, Emory University, Atlanta, GA 30322, USA
2 Department of Computer Science, Emory University, Atlanta, GA 30322, USA
* Corresponding author: yana.bromberg@emory.edu, prabakaran@emory.edu

Supplementary Online Material

Supplementary Text

The definition of Orphan siblings

In this work, a protein pair is defined as functionally similar or sibling pair based on the SNN+TM method (Mahlich, et al., 2023), i.e. structural similarity as defined by TM score ≥0.7 and the SNN functional similarity score ≥0.98. The dataset of proteins used in our study is subset of proteins in the MGnify database of metagenome-assembled genomes (Mitchell, et al., 2020) with <30% sequence identity with UniRef proteins (Suzek, et al., 2015). In other words, our dataset consists of 11,444 orphan proteins with no homology to any annotated proteins in existing databases. The following pseudo-code explains the protocol used to annotate protein pairs as siblings. The respective code has been deposited at https://bitbucket.org/bromberglab/siblings-detector/.

Steps to identify siblings

```
Step 1: Compute TM score
a) Fetch ESM predicted structure for each protein[a]
b) Filter for structures with pLDDT and pTM > 0.90
c) Align structures with each other using FoldSeek(van Kempen, et
   al., 2022)
d) Compute TM scores
Step 2: Compute SNN score
a) Fetch gene sequence for each protein
b) Calculate SNN score for each gene pair

Step 3: Annotated Orphan siblings
For each protein pair with TM score and SNN score
    If TM score ≥ 0.7 and SNN score ≥ 0.98 Then
       Annotate as "sibling"
    Else
       Annotate as "unlabelled"/"non-sibling"
    EndIf
```

[a]ESM structures can be fetched from ESM atlas or predicted using ESMFold (Lin, et al., 2022).

Tables

Table S1: Evaluation of experimental enzyme annotation as "ideal predictor"

Ratio of siblings to non-siblings	# of siblings	# of non-siblings	ΔS	Precision	Recall	Specificity	F1 score	Accuracy	Balanced Accuracy	MCC
49:1	1927	39	0.739	0.999	0.879	0.963	0.935	0.880	0.921	0.340
9:1	1927	214	0.736	0.995	0.879	0.959	0.933	0.887	0.919	0.622
4:1	1927	481	0.736	0.989	0.879	0.960	0.930	0.895	0.919	0.739
**1:1**	**1927**	**1927**	**0.737**	**0.957**	**0.879**	**0.960**	**0.916**	**0.919**	**0.919**	**0.842**
1:4	1927	7708	0.737	0.848	0.879	0.961	0.863	0.944	0.920	0.828
1:9	1927	17343	0.737	0.711	0.879	0.960	0.786	0.952	0.919	0.765
1:49	1927	94423	0.737	0.312	0.879	0.960	0.460	0.959	0.919	0.509
1:99	1927	190773	0.737	0.183	0.879	0.960	0.303	0.960	0.919	0.390
1:499	1927	961573	0.737	0.043	0.879	0.960	0.081	0.960	0.919	0.188

We used experimental annotation as the ground truth function terms and evaluated these annotations for enzymes in UniProt database through our proposed SNN+TM approach. Values listed in the above table reflect the average performance of the ground truth annotations over 100 iterations.

Table S2: Evaluation of a random baseline classifier

**Ratio of siblings to non-siblings**	**# of siblings**	**# of non-siblings**	**ΔS**	***ROC AUC***	***PR AUC***	**Precision**	**Recall**	**Specificity**	**F1 score**	***F1 max***	**Accuracy**	**Balanced Accuracy**	**MCC**
49:1	1927	39	0.000	0.500	0.980	0.980	0.500	0.499	0.662	0.990	0.500	0.500	0.000
9:1	1927	214	0.000	0.501	0.900	0.901	0.502	0.502	0.644	0.947	0.502	0.502	0.002
4:1	1927	481	0.001	0.501	0.801	0.800	0.501	0.499	0.616	0.889	0.500	0.500	0.000
**1:1**	**1927**	**1927**	**0.000**	**0.501**	**0.501**	**0.501**	**0.500**	**0.501**	**0.500**	**0.667**	**0.501**	**0.501**	**0.002**
1:4	1927	7708	-0.001	0.499	0.200	0.199	0.499	0.500	0.285	0.334	0.499	0.499	-0.001
1:9	1927	17343	0.000	0.500	0.100	0.100	0.500	0.500	0.167	0.182	0.500	0.500	0.000
1:49	1927	94423	0.000	0.500	0.020	0.020	0.500	0.500	0.038	0.040	0.500	0.500	0.000
1:99	1927	190773	0.000	0.500	0.010	0.010	0.500	0.500	0.020	0.020	0.500	0.500	0.000
1:499	1927	961573	0.000	0.500	0.002	0.002	0.500	0.500	0.004	0.004	0.500	0.500	0.000

Values listed in the above table reflect the average performance of a random baseline classifier over 100 iterations (See **Table S1** for details).

Table S3: **List of methods**

S. No	Method	Reference	Source	Type
1	DeepFRI	(Gligorijevic, et al., 2021)	https://beta.deepfri.flatironinstitute.org/	Webserver
2	ECPred	(Dalkiran, et al., 2018)	https://ecpred.kansil.org/	Standalone
3	GhostKOALA	(Kanehisa, et al., 2016)	https://www.kegg.jp/ghostkoala/	Webserver
4	GOPredSim	(Littmann, et al., 2021)	https://github.com/Rostlab/goPredSim	Python package
5	HMMER	(Potter, et al., 2018)	http://hmmer.org/	Standalone
6	Mantis	(Queiros, et al., 2021)	https://github.com/PedroMTQ/mantis	Standalone
7	NetGO	(Yao, et al., 2021)	https://issubmission.sjtu.edu.cn/ng2/	WebServer
8	ProtCNN	(Bileschi, et al., 2022)	https://github.com/google-research/google-research/tree/master/using_dl_to_annotate_protein_universe	Python package
9	ProteInfer	(Sanderson, et al., 2021)	https://github.com/google-research/proteinfer	Python package
10	Bepler	(Bepler and Berger, 2019)	https://github.com/sacdallago/bio_embeddings	Python package
11	CPCProtEmbedder	(Lu, et al., 2020)	https://github.com/sacdallago/bio_embeddings	Python package
12	ESM	(Lin, et al., 2022)	https://github.com/sacdallago/bio_embeddings	Python package
13	LookingGlass	(Hoarfrost, et al., 2022)	https://github.com/ahoarfrost/LookingGlass	Python package
14	ProtT5	(Elnaggar, et al., 2020)	https://github.com/sacdallago/bio_embeddings	Python package
15	SeqVec	(Heinzinger, et al., 2019)	https://github.com/sacdallago/bio_embeddings	Python package
16	Word2Vec	(Dallago, et al., 2021)	https://github.com/sacdallago/bio_embeddings	Python package

Table S4: **Evaluation on a dataset of single domain proteins**

Tool	Term	ΔS	$F1_{max}$	PR AUC	ROC AUC	Precision	Recall
ECPred	EC1	**0.21**	**0.69**	**0.75**	**0.61**	**0.56**	**0.93**
ECPred	EC3	**0.15**	**0.68**	**0.74**	**0.58**	**0.55**	**0.92**
Mantis	EC1	0.06	0.63	0.68	0.56	0.53	0.81
Mantis	EC3	0.13	0.62	0.64	0.60	0.59	0.68
DeepFRI	GO	0.06	0.46	0.62	0.64	0.64	0.39
GOPredSim	GO	**0.39**	**0.75**	**0.79**	**0.75**	**0.67**	**0.88**
Mantis	GO	0.12	0.61	0.62	0.60	0.57	0.70
NetGO	GO	0.03	**0.53**	0.55	0.55	0.52	0.67
ProteInfer	GO	0.02	0.33	0.44	0.55	0.62	0.24
GhostKOALA	KO	**0.03**	**0.34**	**0.45**	**0.55**	**0.65**	**0.25**
Mantis	COG	0.03	0.49	0.50	0.53	0.54	0.47
HMMER	Pfam	**0.07**	**0.63**	**0.69**	**0.54**	**0.53**	**0.83**
Mantis	Pfam	0.03	0.53	0.54	0.53	0.53	0.55
ProtCNN	Pfam	0.00	0.11	0.34	0.51	0.62	0.07

We evaluated the performance of the methods in a subset of our original test dataset, consists of only proteins with sequence length less than or equal to 150 residues. The dataset consists of 1700 siblings among 4376 protein pairs. Values listed are average over 100 iterations of under sampling (**Methods**). Performance of the selected, top-performing methods is identified in bold.

Table S5: **Characteristics of protein pairs predicted as siblings**

	Range	Total Orphan pairs	Siblings	ECPred	GOPredSim	NetGO	HMMER	GhostKOALA	SwiftOrtho
Sequence Identity	<40%	99.32	89.66 --	96.48 --	98.6--	98.97--	98.48--	96.61--	52.12--
	40-60%	0.37	5.05++	0.44	0.29	0.56++	0.81++	1.91++	24.7++
	60-80%	0.23	3.76++	2.2++	0.85++	0.35++	0.53++	1.27++	17.38++
	80-90%	0.08	1.53++	0.88+	0.26+	0.12++	0.17+	0.21	5.79++
TM-score	0-0.5	14.55	0.0--	9.69-	7.97--	12.69--	8.2--	2.12--	2.86--
	0.5-0.7	45.07	0.0--	48.9	40.96--	43.81--	37.19--	3.18--	6.04--
	0.7-1.0	40.38	100.0++	41.41	51.07++	43.51++	54.61++	94.7++	91.1++
SNN score	0-0.5	50.12	0.0--	38.77--	52.16+	48.71--	52.72++	63.14++	34.81--
	0.5-0.98	45.81	0.0--	52.86+	43.53-	46.46++	42.21--	35.38--	44.63
	0.98-1.0	4.07	100.0++	8.37+	4.32	4.83++	5.07++	1.48-	20.56++

Values listed are the percentages of predicted siblings in the given range of sequence identity, TM-score and SNN score. The values were compared against the corresponding counts in the entire dataset of orphan pairs via the two-sided Fischer's exact test. +/- denotes a significant increase/decrease with p-value in [0.001, 0.05] range, while ++/-- denotes p-value of < 0.001

Table S6: **Characteristics of protein pairs predicted as siblings**

	Range	Total Orphan pairs	Siblings	ECPred	GOPredSim	NetGO	HMMER	GhostKOALA	SwiftOrtho
Sequence Identity	<40%	307,434	5,576	9/219	79/3,724	4,592/200,223	347/10,696	3/456	242/2,150
	40-60%	1,155	314	1/1	2/11	302/1,131	19/88	1/9	259/1,019
	60-80%	720	234	4/5	7/32	233/714	20/58	2/6	232/717
	80-90%	240	95	1/2	2/10	95/237	12/19	0/1	95/239
Total		309,549	6,219	15/227	90/3,777	5,222/202,305	398/10,861	6/472	828/4,125

*Values listed are the counts of correctly predicted siblings of total identified by the method as siblings in the given range of sequence identity.

Figures

SI Fig. 1

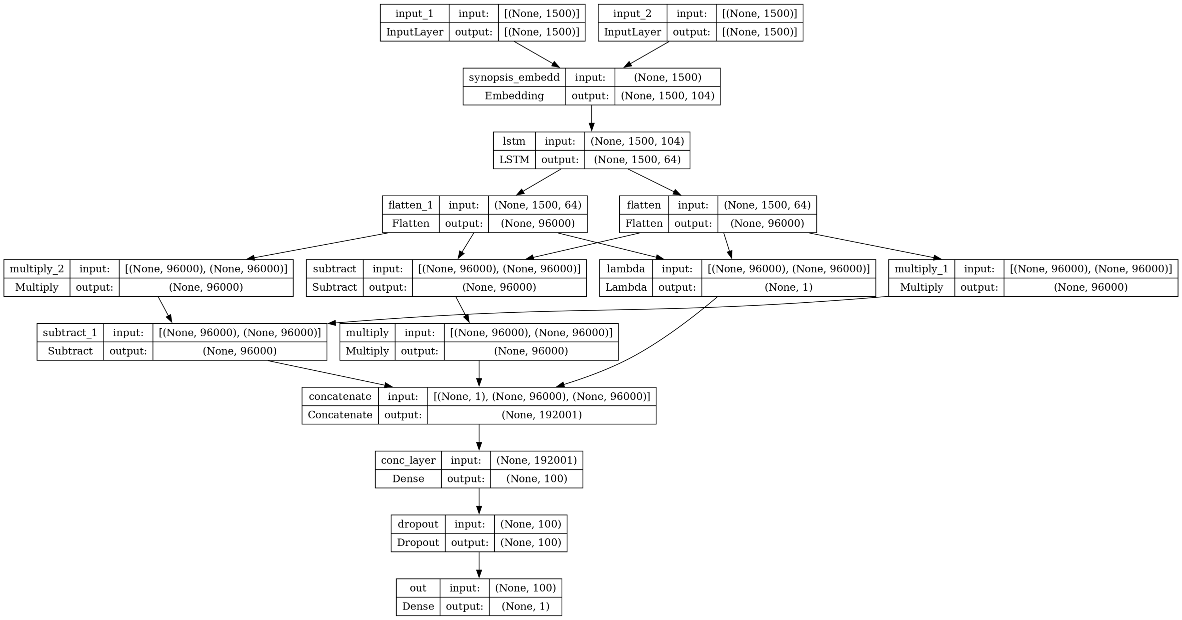

SI Figure 1: Architecture of the Siamese Neural Network (SNN). The flowchart describes the architecture of the Siamese Neural Network (SNN) model used in this study. The SNN architecture includes 1) the pretrained embedding layer from LookingGlass (Hoarfrost, et al., 2022), 2) an LSTM layer and 3) computation of distance between embeddings. The input consists of two protein-encoding genes, is split into two sequences of codons, and converted into embeddings through the LookingGlass embedding layer. Then the two sequences of embeddings are propagated through a layer of LSTM. Finally, the similarity between the two embeddings is computed as a combination of difference and cosine distance between embeddings and passed through a dense layer followed by sigma activation, which produces a similarity score.

SI Fig. 2

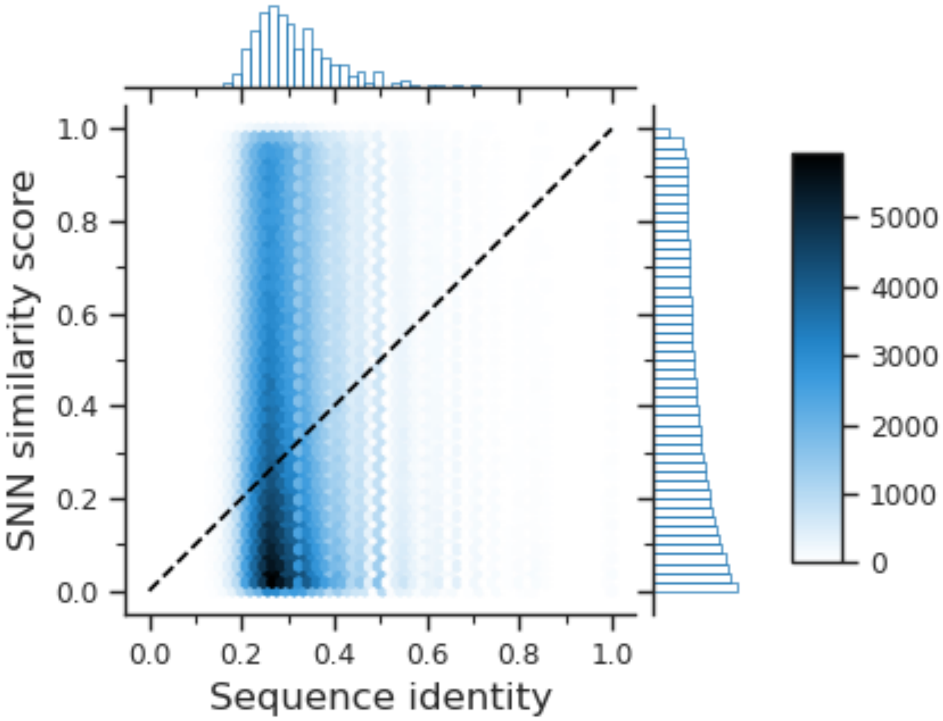

SI Figure 2. Correlation between SNN score and Sequence identity. The plot shows the distribution of sequence identities vs. SNN scores of 1,745,646 enzyme pairs used to validate our approach.

SI Fig. 3

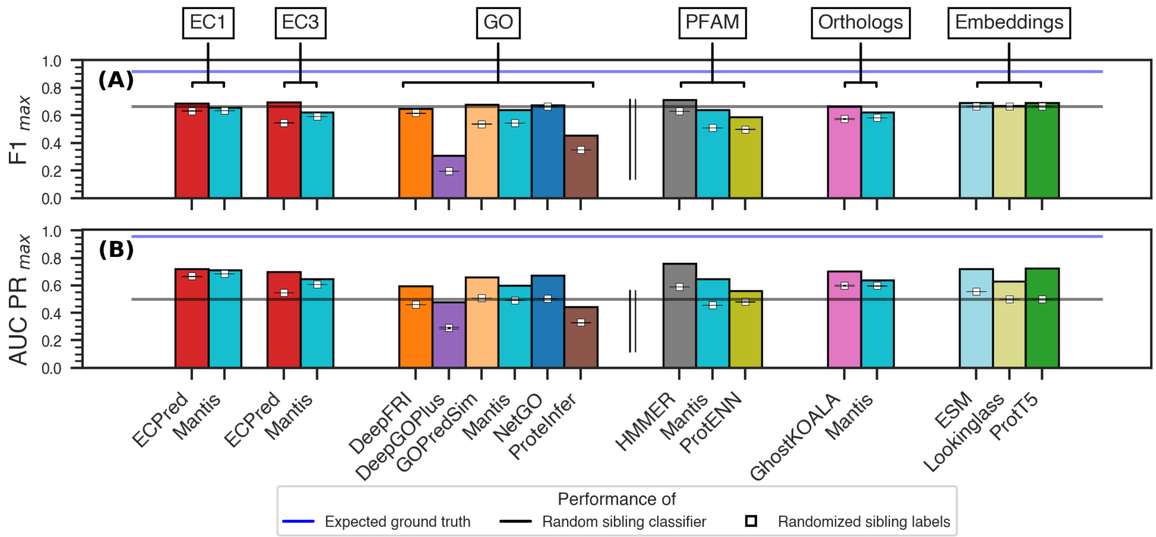

SI Figure 3. Comparing method performance across embeddings. The bar plots show the highest method (A) F1 scores, and (B) Area under the Precision-Recall curves for the prediction of orphan siblings (TM-score and SNN cutoffs of 0.7 and 0.98, respectively). The gray lines indicate the performance of the random classifier.

SI Fig. 4

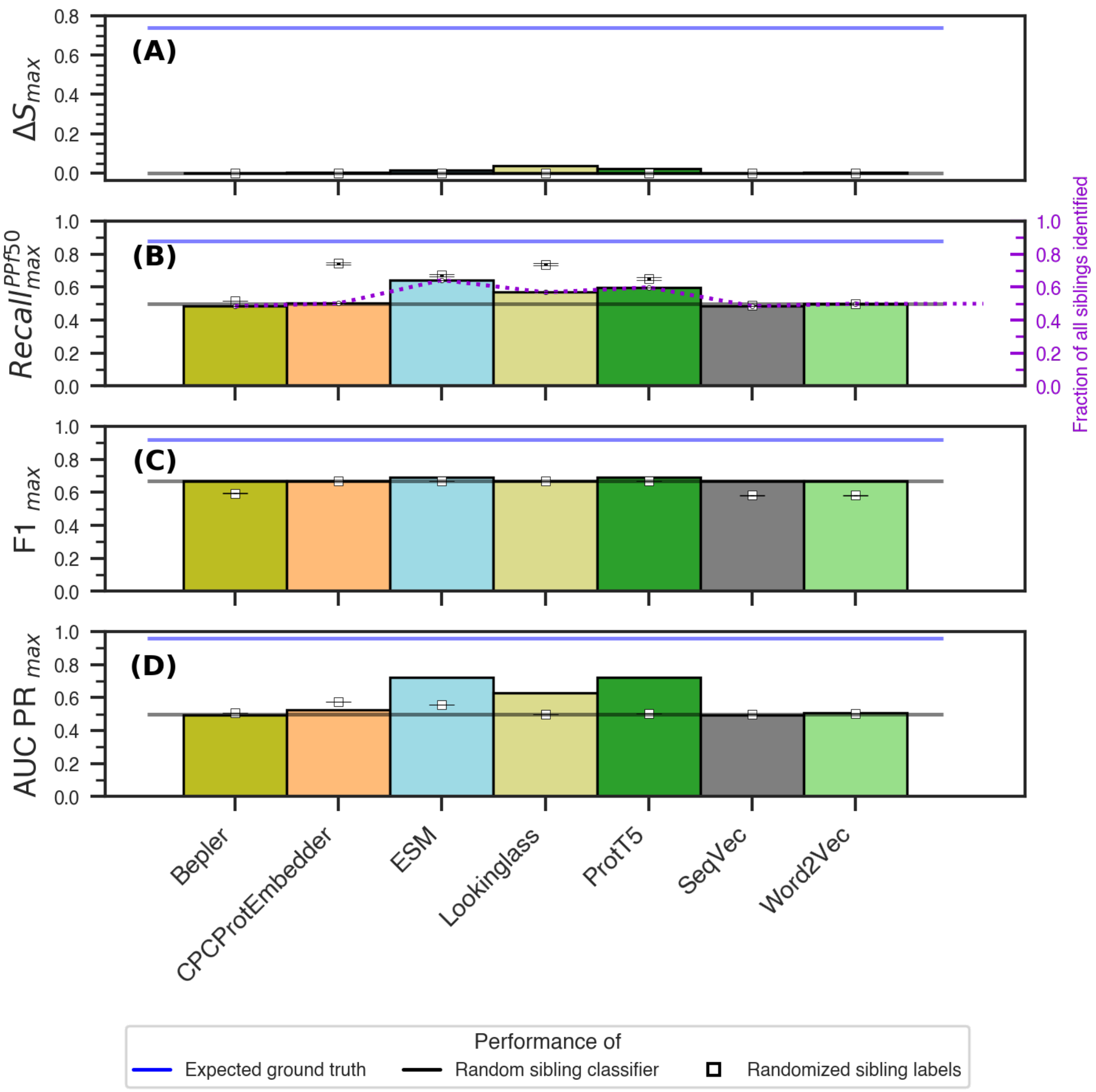

SI Figure 4. Comparing method performance across embeddings. The bar plots show the highest(max) method (A) ΔS, (B) $Recall^{PPf50}_{max}$, (C) F1 scores, and (D) Area under the Precision-Recall curves for the prediction of orphan siblings (TM-score and SNN cutoffs of 0.7 and 0.98, respectively). The gray lines indicate the performance of the random baseline classifier.

SI Fig. 5

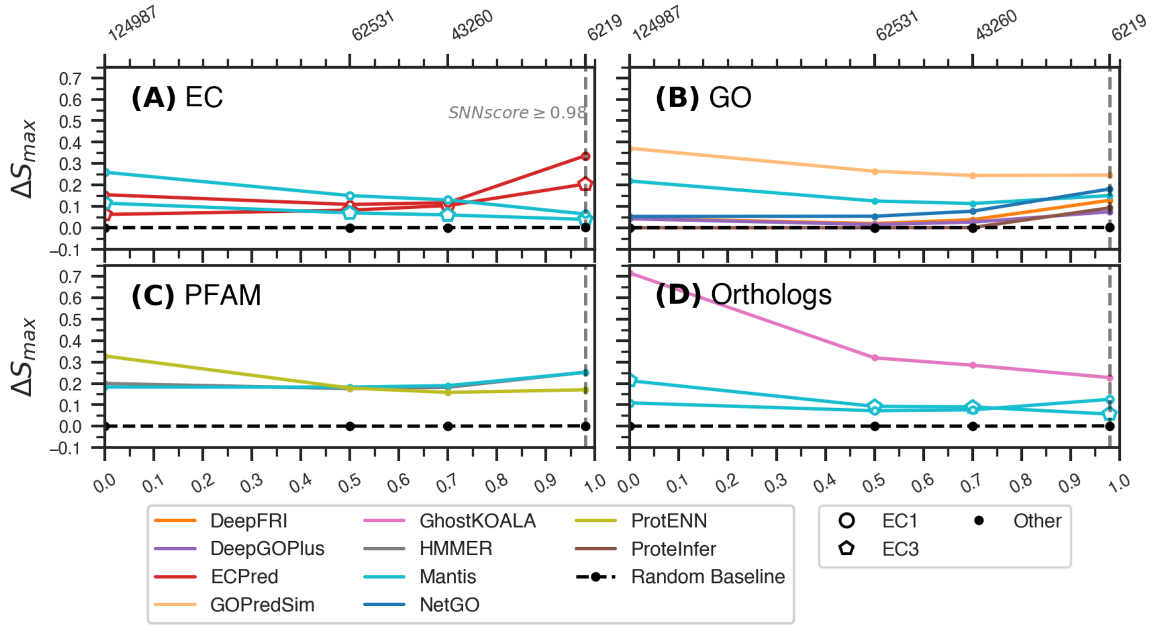

SI Figure 5. Variations in ΔSimilarity score over SNN-score cutoffs. The highest ΔS were computed at TM-score cutoff of 0.7 and at different SNN score cutoffs [0, 0.5, 0.7, 0.98] by varying the prediction score threshold. The average performance of 100 random baseline classifiers is plotted for comparison in each panel (black line).

SI Fig. 6

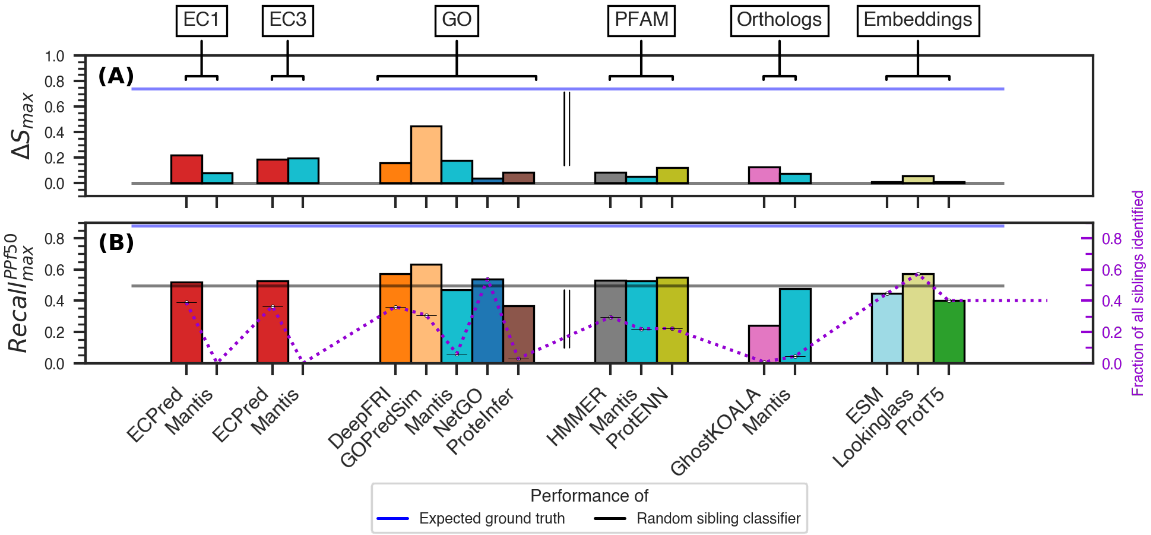

SI Figure 6: Comparing method performance in each annotation category. The bar plots show the highest (max) method **(A)** ΔS, and **(B)** $Recall^{PPf50}_{max}$ for the prediction of orphan siblings (TM-score and SNN cutoffs of 0.7 and 0.98, respectively), based on: Enzyme Commission numbers (EC1 – first digit, EC3 – up to third digit), Gene Ontology Molecular Function terms, Pfam domains, Ortholog groups and language model embeddings. The highest scores were selected for each method by varying the prediction score threshold. The double line separates explicit functional annotations (EC and GO) from Pfam and Ortholog definitions and embedding similarity. The gray lines indicate the average performance of 100 random baseline classifiers. The blue lines indicate the expected performance of the ground-truth annotation as observed in Table S1. Note DeepGOPlus was removed due to absence of sufficient protein pairs with predicted annotations.

SI Fig. 7

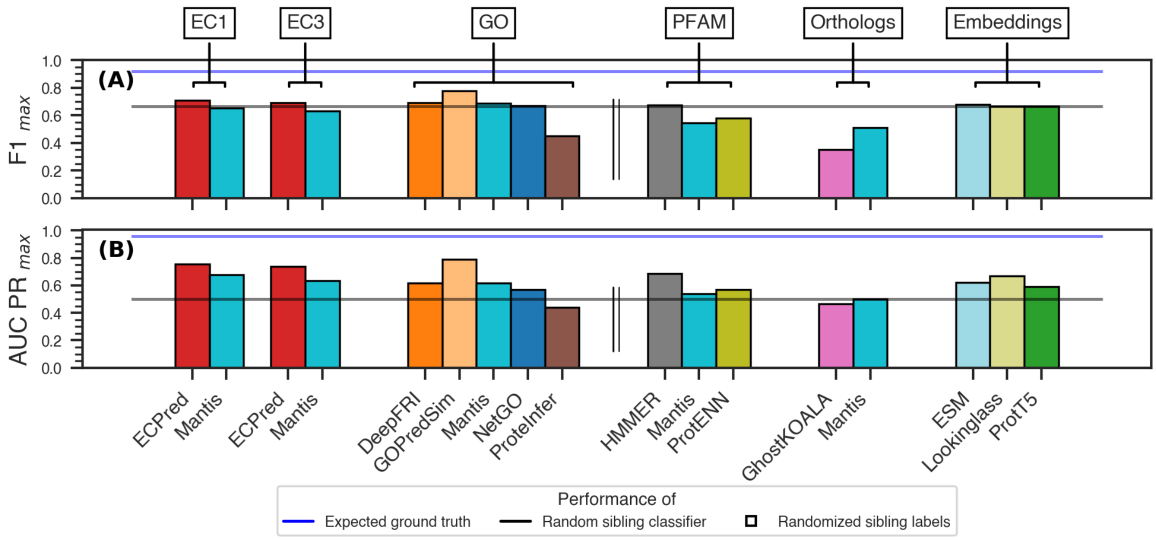

SI Figure 7: Comparing method performance in each annotation category. The bar plots show the highest (max) method **(A)** F1 scores, and **(C)** Area under the Precision-Recall curves for the prediction of orphan siblings (TM-score and SNN cutoffs of 0.7 and 0.98, respectively), based on: Enzyme Commission numbers (EC1 – first digit, EC3 – up to third digit), Gene Ontology Molecular Function terms, Pfam domains, Ortholog groups and language model embeddings. The highest scores were selected for each method by varying the prediction score threshold. The double line separates explicit functional annotations (EC and GO) from Pfam and Ortholog definitions and embedding similarity. The gray lines indicate the average performance of random baseline classifiers. The blue lines indicate the expected performance of the ground-truth annotation as observed in Table S1.

References:

Bepler, T. and Berger, B. Learning protein sequence embeddings using information from structure. *ArXiv* 2019;abs/1902.08661.

Bileschi, M.L., *et al.* Using deep learning to annotate the protein universe. *Nature Biotechnology* 2022;40(6):932-937.

Dalkiran, A., *et al.* ECPred: a tool for the prediction of the enzymatic functions of protein sequences based on the EC nomenclature. *BMC Bioinformatics* 2018;19(1):334.

Dallago, C., *et al.* Learned Embeddings from Deep Learning to Visualize and Predict Protein Sets. *Curr Protoc* 2021;1(5):e113.

Elnaggar, A., *et al.* ProtTrans: Towards Cracking the Language of Life's Code Through Self-Supervised Deep Learning and High Performance Computing. *bioRxiv* 2020.

Gligorijevic, V., *et al.* Structure-based protein function prediction using graph convolutional networks. *Nat Commun* 2021;12(1):3168.

Heinzinger, M., *et al.* Modeling aspects of the language of life through transfer-learning protein sequences. *BMC Bioinformatics* 2019;20(1):723.

Hoarfrost, A., *et al.* Deep learning of a bacterial and archaeal universal language of life enables transfer learning and illuminates microbial dark matter. *Nat Commun* 2022;13(1):2606.

Kanehisa, M., Sato, Y. and Morishima, K. BlastKOALA and GhostKOALA: KEGG Tools for Functional Characterization of Genome and Metagenome Sequences. *J Mol Biol* 2016;428(4):726-731.

Lin, Z., *et al.* Evolutionary-scale prediction of atomic level protein structure with a language model. *bioRxiv* 2022:2022.2007.2020.500902.

Littmann, M., *et al.* Embeddings from deep learning transfer GO annotations beyond homology. *Scientific Reports* 2021;11(1):1160.

Lu, A.X., *et al.* Self-Supervised Contrastive Learning of Protein Representations By Mutual Information Maximization. *bioRxiv* 2020:2020.2009.2004.283929.

Mahlich, Y., *et al.* Learning from the unknown: exploring the range of bacterial functionality. *Nucleic Acids Res* 2023;51(19):10162-10175.

Mitchell, A.L., *et al.* MGnify: the microbiome analysis resource in 2020. *Nucleic Acids Res* 2020;48(D1):D570-D578.

Potter, S.C., *et al.* HMMER web server: 2018 update. *Nucleic Acids Res* 2018;46(W1):W200-W204.

Queiros, P., *et al.* Mantis: flexible and consensus-driven genome annotation. *Gigascience* 2021;10(6).

Sanderson, T., *et al.* ProteInfer: deep networks for protein functional inference. *bioRxiv* 2021:2021.2009.2020.461077.

Suzek, B.E., *et al.* UniRef clusters: a comprehensive and scalable alternative for improving sequence similarity searches. *Bioinformatics* 2015;31(6):926-932.

van Kempen, M., *et al.* Foldseek: fast and accurate protein structure search. *bioRxiv* 2022.

Yao, S., *et al.* NetGO 2.0: improving large-scale protein function prediction with massive sequence, text, domain, family and network information. *Nucleic Acids Res* 2021;49(W1):W469-W475.
